# Muscle- and Gland-Specific Dosing of Botulinum Toxin in Facial Aesthetics: Review of Dose Ranges, Target Depth, Muscle Thickness, and Neuromuscular or Glandular Anatomy

**DOI:** 10.3390/toxins18100444

**Published:** 2026-10-14

**Authors:** Kyu-Ho Yi, Kyu-Lim Lee

**Affiliations:** 1 You and I Clinic, Seoul 06123, Republic of Korea; kyuho90@daum.net; 2 Division in Anatomy and Developmental Biology, Department of Oral Biology, Human Identification Research Institute, BK21 FOUR Project, Yonsei University College of Dentistry, Seoul 03722, Republic of Korea

**Keywords:** botulinum neurotoxin type A, facial muscles, muscle-specific dosing, muscle thickness, target depth, neuromuscular junction, ultrasound-guided injection, masseter, temporalis, salivary glands

## Abstract

Botulinum neurotoxin type A (BoNT-A) dosing in facial aesthetics varies across anatomical targets, formulations, and treatment indications. This structured narrative review examined published dose ranges and the potential relevance of muscle thickness, target depth, functional anatomy, and injection localization to individualized treatment planning. English-language regulatory, clinical, anatomical, ultrasound, and consensus literature available through 13 April 2026 was considered. Evidence was interpreted according to source type, distinguishing approved product-specific regimens, controlled clinical studies, observational reports, and anatomy- or expert-derived recommendations. Muscle bulk may inform masseter contouring, but the literature does not establish a validated thickness-based dosing equation applicable across facial muscles. For facial expression muscles, contraction patterns, treatment objectives, and preservation of adjacent function are central considerations. Evidence for aesthetic dosing of the temporalis and salivary glands remains limited, and glandular indications require separate assessment from skeletal muscle treatment. Anatomically informed placement has a plausible mechanistic rationale, whereas evidence that motor-zone targeting consistently reduces toxin requirements is insufficient. Ultrasound may assist selected anatomically complex or previously treated cases but is not established as necessary for routine injections. Safe dose selection requires formulation-specific interpretation, indication-specific evidence, individualized anatomical assessment, and recognition of the limitations of existing studies.

## 1. Introduction

Botulinum neurotoxin type A has become the dominant neuromodulator for the treatment of dynamic rhytids, brow imbalance, lower-face depressor overactivity, platysmal banding, and masseteric hypertrophy. It is used in on-label upper-face indications such as glabellar lines, forehead lines, and lateral canthal lines, but it is also widely used off-label in the nose, upper lip, perioral region, chin, jawline, neck, and lower facial contouring [1,2,3,4,5,6]. The breadth of indications has expanded faster than the dose literature. As a result, many injectors learn conventional dosing patterns for individual regions, such as 20 units for the glabella, 20 units for the forehead when treated with the glabella, 24 units for crow’s feet, a few units for the DAO, several units for the mentalis, and larger totals for the masseter, without necessarily understanding why different muscles require such different dosing approaches [4,6,7,8,9,10].

The mechanism of BoNT-A suggests that dosing should never be treated as a purely cosmetic ritual. After binding to high-affinity receptors at the cholinergic presynaptic terminal, the toxin undergoes internalization and cleavage of soluble N-ethylmaleimide-sensitive factor attachment protein receptor (SNARE) proteins, functionally blocking acetylcholine release at the neuromuscular junction and producing a temporary chemical denervation of the target muscle [2,3]. The effect is local rather than diffuse across the entire face, but it is not confined to an infinitesimal point. Clinical response depends on the balance between placement precision, local concentration, injection volume, depth, tissue plane, and the arrangement of the motor endplates that are exposed to the injected toxin [11,12,13,14]. A muscle can therefore be undertreated because too few units were used, but it can also be undertreated because an adequate number of units was placed in the wrong plane, too far from the active motor zone, or into a compartment separated by tendon or fascial barriers [13,14,15,16,17,18,19].

This mechanistic perspective is one reason the concept of “dose per muscle” is more useful than “dose per wrinkle.” A glabellar wrinkle does not behave like a crow’s feet pattern, a perioral line, or a platysmal band, because the underlying anatomy is fundamentally different. The corrugator and procerus are small but deep and intimately related to brow position. The frontalis is broad, thin, and functionally essential for brow elevation. The lateral orbicularis oculi are superficial and radially oriented, with an adjacent risk zone for unwanted diffusion into smile elevators or extraocular structures. The DAO, mentalis, and orbicularis oris are delicate lower-face muscles whose overtreatment can cause conspicuous functional asymmetry. By contrast, the masseter is a thick three-dimensional masticatory muscle that is often treated not just for movement, but for bulk reduction and facial contouring [4,6,7,9,10,15,16,17,18,20,21,22,23,24,25,26,27,28,29,30].

Although most aesthetic BoNT-A targets are muscles, selected glandular structures also influence lower- and lateral-face contour. The parotid and submandibular glands are increasingly examined as BoNT targets in sialorrhea, benign glandular hypertrophy, and gland-related facial fullness. Their dosing logic is different from muscle dosing because the desired effect is reduced secretion and glandular prominence rather than weakening of contraction [31,32].

The literature also faces a definitional problem. “Dose” is often discussed as if botulinum toxin units were interchangeable between products, but they are not. The potency assays for onabotulinumtoxinA, incobotulinumtoxinA, abobotulinumtoxinA, prabotulinumtoxinA, and letibotulinumtoxinA are not standardized to a common biological unit, and the available evidence does not support a universal conversion ratio that can be safely applied across all indications [1]. In aesthetic practice, many clinicians use pragmatic rules of thumb, but those rules do not eliminate product-specific differences in concentration, spread, reconstitution preferences, labeling history, and the way dose–response is expressed in published trials [1,5,33]. A paper on “dose for the corrugator” is therefore only clinically meaningful if it identifies which product was used or if it explicitly normalizes discussion to one reference formulation, usually onabotulinumtoxinA.

Another important unresolved question is how much muscle thickness should influence dose. In routine practice, clinicians often assume that larger, stronger, or thicker muscles require more toxin. That assumption is intuitively correct in some situations, especially in masseteric hypertrophy. Yet the relationship is far less straightforward in thin facial muscles. A strong frontalis may require a conservative dose if the patient relies on it to support the brow. Thin perioral muscles may still require only small doses because their therapeutic window is narrow. A thicker corrugator can produce severe vertical glabellar lines, but inaccurate depth or excessive medial diffusion can still create ptosis even with a moderate dose [4,5,6,7,20,21]. Thickness, in other words, is not irrelevant, but it is not equivalent to safe dose capacity.

In parallel with these questions, a second line of evidence has become increasingly influential: the importance of injecting close to neuromuscular junction-rich zones or intramuscular neural arborization areas. Therapeutic and experimental studies outside pure aesthetics show that BoNT effect increases when the toxin is delivered closer to the endplate zone and falls when the same dose is placed farther away [11,12,13]. This observation has profound consequences for facial aesthetics. If part of the required dose is compensating for imperfect placement, then better placement may allow similar results with fewer units, fewer touch-ups, or less diffusion into adjacent muscles. The recent spread of high-resolution ultrasound in aesthetic practice has made this question clinically relevant rather than merely theoretical [7,16,17,19,34,35,36].

The present review was therefore developed around four practical questions. First, what dose ranges are supported by the current literature for the major facial muscles used in cosmetic BoNT-A treatment? Second, how should these doses be interpreted across different muscles and formulations? Third, does muscle thickness correlate with dose in a clinically meaningful way? Fourth, can NMJ-targeted or distributed motor-zone injection improve efficiency enough to reduce total units? The goal is not to provide a rigid universal chart, because such a chart would ignore the variability of facial anatomy and the non-interchangeability of products. Rather, the aim is to build a structured, evidence-based dosing framework that helps the reader understand why some muscles tolerate broad dose ranges whereas others demand tiny increments and great anatomical precision.

A final reason this topic matters is safety. Most clinically significant adverse events in aesthetic BoNT-A therapy are not systemic toxicities; they are local failures of anatomy, dosing logic, or diffusion control. Eyelid ptosis, brow flattening, smile asymmetry, lip incompetence, speech alteration, dysphagia, paradoxical bulging, and impaired mastication are all examples of what can happen when a dose is selected without adequate respect for target depth, muscle function, or adjacent anatomy [4,8,9,26,37,38,39]. A muscle-specific review is therefore not only about efficacy; it is also about understanding where the therapeutic window is wide, where it is narrow, and where better targeting may be more important than escalating units.

## 2. Results

### 2.1. Overview of the Evidence Landscape

The retrieved literature does not support a single universal dosing rule across facial botulinum neurotoxin type A (BoNT-A) targets. Instead, the evidence varies according to the anatomical target, treatment indication, toxin formulation, and study design. For clinical interpretation, the targets can be organized into four indication-related groups. The first comprises established aesthetic indications involving the glabellar complex, forehead, and lateral canthal region, for which regulatory labeling and controlled clinical investigations provide relatively standardized formulation-specific treatment regimens [4,5,8,9,20,21,33,40,41]. The second includes predominantly off-label aesthetic applications involving perinasal, perioral, selected periocular, and lower-face mimetic muscles, where anatomical investigations, expert consensus, and smaller clinical studies contribute substantially to dose selection [6,10,22,23,24,25,42,43,44,45,46,47]. The third encompasses facial and cervical contouring, particularly treatment of masseteric hypertrophy and platysmal prominence, for which clinical outcomes depend on both muscular activity and changes in contour or muscle volume [15,16,17,18,26,27,28,29,30,36,37,48]. The fourth includes accessory masticatory and glandular targets, notably the temporalis, parotid gland, and submandibular gland, where functional treatment objectives, anatomical localization, and the distinction between muscular and secretory mechanisms require separate interpretation [31,32,49,50,51,52]. These indication-related groups should not be confused with the four evidence-source categories used in Table 1, which separately distinguish regulatory labeling, randomized or prospective clinical evidence, observational or case-based evidence, and the authors’ proposed synthesis.

Several anatomical and clinical principles recur across these indication groups, although the strength of supporting evidence is not uniform. First, the distribution of a formulation-specific total dose may influence treatment response through differences in target coverage, injection volume, tissue plane, and proximity to motor endplate-rich regions [9,13,14,21,24,26,36]. However, anatomical plausibility and experimental evidence should not be interpreted as proof that a particular distribution strategy consistently achieves equivalent clinical efficacy with fewer units across all facial targets. Second, the functional consequences of unintended muscle weakening differ substantially by region. In the lower face, even modest off-target effects involving the depressor anguli oris, depressor labii inferioris, mentalis, or orbicularis oris may produce visible asymmetry or interfere with oral function [6,22,23,24,25]. Consequently, conservative, indication-specific dose selection is especially important in these regions. Third, baseline thickness and muscle volume are more directly relevant to treatments intended to reduce muscular bulk, particularly masseter contouring, than to superficial wrinkle reduction. Nevertheless, thickness alone cannot establish an appropriate dose or replace assessment of muscle function, previous treatment, and adjacent anatomical structures [7,15,16,17,18,27,28,29,30].

**Figure 1:**
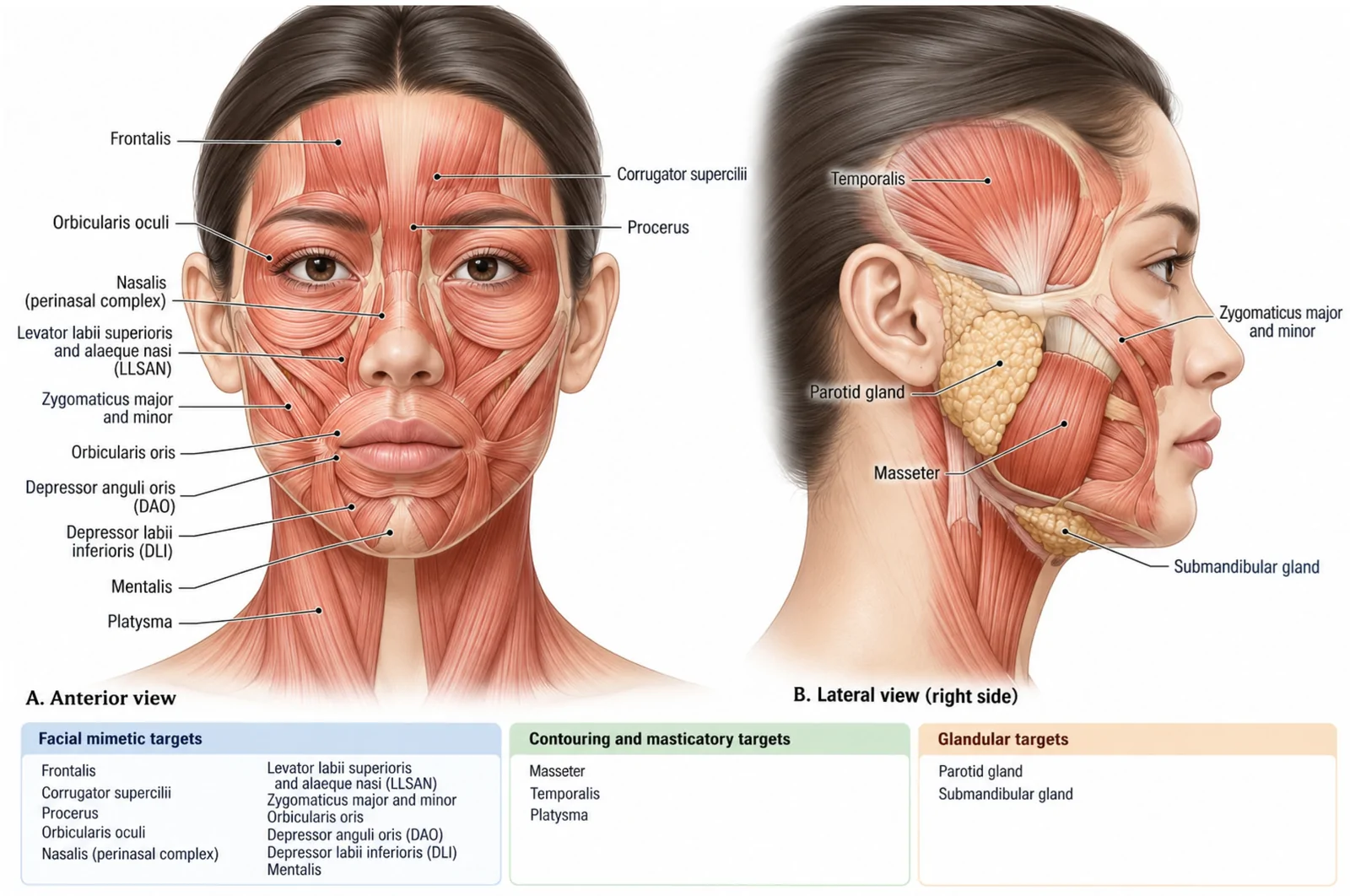
Anatomical overview of the principal muscular and glandular targets discussed in this review. (**A**) Anterior view illustrating the major facial mimetic muscles, including the frontalis, corrugator supercilii, procerus, orbicularis oculi, nasalis and perinasal complex, levator labii superioris alaeque nasi (LLSAN), zygomaticus major and minor, orbicularis oris, depressor anguli oris (DAO), depressor labii inferioris (DLI), mentalis, and platysma. (**B**) Right lateral view highlighting the temporalis and masseter muscles, together with the parotid and submandibular glands. These anatomical structures represent distinct targets considered in facial botulinum neurotoxin type A (BoNT-A) treatment, encompassing aesthetic, functional, contouring, and glandular applications. Differences in anatomical location, muscle architecture, tissue depth, and functional characteristics are relevant to target-specific dose selection and safety considerations. The illustration is schematic and is not intended to indicate specific injection points, doses, or treatment depths.

The temporalis and salivary gland literature further illustrates the importance of distinguishing treatment mechanisms and indications. The temporalis is a masticatory muscle whose size and activity may be relevant to selected functional or contour-related presentations, including compensatory recruitment after masseter treatment. By contrast, the parotid and submandibular glands are secretory structures, and their BoNT-A treatment is primarily supported by functional salivary indications and anatomical localization studies rather than by the same dose–response evidence used for facial mimetic muscles [31,32,49,50,51,52]. Gland dimensions and surrounding structures may influence localization and safety, but gland thickness should not be assumed to determine dose in the same manner as muscle hypertrophy. Accordingly, functional salivary treatment data should not be directly extrapolated to aesthetic glandular contouring. The principal facial mimetic, masticatory, cervical, and glandular anatomical targets discussed in this review are illustrated in Figure 1.

### 2.2. Product Non-Interchangeability as a Prerequisite for Dose Interpretation

Before considering any muscle-specific recommendations, it is necessary to restate that BoNT units are product-specific [1]. OnabotulinumtoxinA is the most useful reference point for literature synthesis because many studies, the historic U.S. labeling pathway, and a large body of full-face practice patterns are anchored to this formulation. However, even when approximate clinical conversion customs exist, they should not be mistaken for validated equivalence across every indication or muscle. A dose of 20 units in the glabella means something very specific if it refers to onabotulinumtoxinA, but it does not become universally transferable merely because a clinician informally uses a ratio to another toxin in practice [1,5,33].

Product non-interchangeability is particularly important when interpreting lower-face, cervical, masticatory, and glandular studies, because the literature includes multiple BoNT-A formulations and frequently reports different dose units, treatment patterns, and clinical endpoints [1,5,10,22,26,33]. For this reason, Table 1 distinguishes doses reported in regulatory labeling and clinical investigations from the authors’ proposed clinical synthesis. OnabotulinumtoxinA is used as the reference formulation when discussing its own labeled regimens or an explicitly onabotulinumtoxinA-oriented synthesis; however, doses originating from studies of other formulations are identified by product and are not converted into presumed equivalent onabotulinumtoxinA units. Regulatory approval is interpreted at the level of the specific product and indication, not as approval of every individual muscle or anatomical target within a treatment region. This distinction is essential when comparing established aesthetic indications with off-label applications and when interpreting differences between clinical trial doses and proposed starting ranges.

### 2.3. The Variables That Modify Dose Across All Muscles

Five interrelated factors modify dose interpretation across facial targets. First, muscle function determines the potential consequences of weakening. For example, frontalis treatment must account for its role in brow elevation, whereas lower-face depressor treatment must consider the balance between opposing muscle groups [4,20,22,23,24,25,40]. Second, muscle geometry and anatomical depth influence target accessibility and treatment distribution; thin superficial muscles and bulky multilayered muscles should not be approached using identical anatomical assumptions [7,9,15,16,17,18,26]. Third, baseline muscle activity, hypertrophy, and the intended outcome influence the relevance of muscle volume to dose selection. This relationship is particularly important for masseter contouring, where reduction in muscle thickness or volume is itself a treatment outcome [15,16,17,18,27,28,29,30]. Fourth, proximity to functionally important adjacent structures affects the consequences of toxin spread and may justify a more conservative approach in anatomically sensitive regions [6,22,23,24,25,26]. Fifth, previous BoNT-A exposure and treatment response may influence subsequent dosing. Repeated treatment of hypertrophic masticatory muscles may produce persistent changes in muscle thickness or volume, so maintenance doses should not automatically reproduce initial doses without reassessment. This consideration is especially relevant to the masseter and may also apply to the temporalis when repeated treatment or compensatory muscular recruitment is present [28,30,49,50,51,52,53].

A sixth consideration is whether imaging can provide clinically useful information beyond conventional anatomical and functional assessment. Ultrasound can visualize variation in muscle thickness, depth, and topography, including differences between individuals and between sides of the same face [7,16,17,19,34,54]. Nevertheless, ultrasound should be regarded as a selective adjunct rather than a universal requirement for facial BoNT-A treatment. Established superficial aesthetic indications can generally be assessed using clinical examination, dynamic facial movement, and anatomical landmarks. Imaging may offer additional value in patients with marked asymmetry, previous treatment-related anatomical changes, uncertain target localization, substantial muscle hypertrophy, or complex glandular anatomy. For repeated masseter or temporalis treatment, ultrasound may help document changes in thickness and guide reassessment of treatment strategy, although thickness measurements alone do not define a validated dose-adjustment algorithm [16,17,18,19,28,30,34,35]. Thus, the rationale for ultrasound should be based on the clinical question and anticipated safety or decision-making benefit, rather than an assumption that imaging is necessary for every injection.

### 2.4. Why Is the Evidence Stronger for Some Muscles than for Others

The certainty with which dose ranges can be interpreted differs substantially among anatomical targets. For established upper-face indications, regulatory labeling and controlled clinical trials provide formulation-specific dosing frameworks, although these frameworks do not necessarily establish the optimal dose for every constituent muscle or individual patient [4,5,8,9,20,21,33,40,41]. For masseter treatment, prospective investigations, randomized studies, and ultrasound-based assessments contribute evidence regarding muscular and contour-related outcomes, but differences in formulation, baseline hypertrophy, outcome measures, and follow-up limit direct comparisons [15,16,17,18,27,28,29,30]. Platysmal treatment is supported by anatomical investigations and controlled clinical trial evidence, including phase 3 evaluation of onabotulinumtoxinA [26,36,37]. In contrast, many perinasal, perioral, and lower-face applications remain dependent on smaller studies, anatomical rationale, and expert consensus, making precise dose–response conclusions less certain [6,10,22,23,24,25,42,43,44,45,46]. The existence of a commonly used clinical dose should therefore not be interpreted as evidence that the dose has been validated in a randomized trial. This distinction is reflected in the separate evidence categories presented in Table 1.

The evidence hierarchy also differs by outcome. For the glabella and forehead, outcome is usually wrinkle severity or patient satisfaction. For platysma prominence, outcome includes severity grading of neck bands and jawline improvement [37]. For masseter treatment, outcome may be ultrasound-measured thickness, lower-face width reduction, bite force change, or patient-reported contouring [16,17,18,27,28,29,30]. For gummy smile, the most objective outcome is millimeters of gingival display reduction [42,43,44]. These different outcome metrics make it impossible to ask one narrow question such as “What is the correct dose for every muscle?” The better question is “What dose range is most consistently associated with an acceptable balance of efficacy and safety for the specific muscular task being treated?” The remainder of the review is organized according to that question.

Table 1 provides a target-specific, evidence-stratified summary of reported BoNT-A doses and the authors’ proposed clinical synthesis. Regulatory label doses are distinguished from doses evaluated in randomized or prospective studies, those described in observational or case-based reports, and evidence-informed starting ranges proposed by the authors. The table also identifies differences in indication, formulation, anatomical considerations, and safety interpretation. Empty or unestablished evidence categories should not be interpreted as evidence of ineffectiveness, but rather as limitations in the dose-specific literature considered. Because BoNT-A potency units are formulation-specific and study outcomes are heterogeneous, the table is intended to support critical interpretation rather than serve as a universal conversion chart or standardized injection protocol [1,5,15,22,33,37].

**Table 1:** Evidence-stratified dose reporting across facial muscular and glandular BoNT-A targets. Regulatory label doses, clinical-study doses, observational/case evidence, and authors’ proposed dose synthesis are reported separately. Proposed ranges are interpretive, not validated universal treatment protocols.

Anatomical Target/Indication	Regulatory Label Dose (BOTOX Cosmetic, U.S.)	Randomized/Prospective Evidence	Observational/Case-Based Evidence	Authors’ Synthesis/Starting Range	Interpretation and Safety
Frontalis—Cosmetic	20 U forehead in 5 sites, administered WITH 20 U glabellar treatment (40 U combined) [55].	Controlled glabellar/forehead clinical literature and dose–response study [8,20,41]; no separately extracted prospective forehead dose.	No independently extracted observational dose; anatomical/clinical discussion [20,54].	10–20 U total	Preserve brow support; brow reserve, forehead height, sex, and compensatory frontalis activity may be more important than thickness.
Corrugator supercilii—Cosmetic	Glabellar complex: 20 U total; 4 U × 2 sites in EACH corrugator plus 4 U procerus; not a standalone corrugator approval [55].	Controlled glabellar clinical evidence [8]; label dose pertains to the entire glabellar complex, not an independent corrugator dose.	Anatomical guidance [21]; no independent observational dose extracted.	6–16 U total for the corrugators; glabellar-complex total commonly 15–25 U	Use small increments. Correct depth and avoidance of diffusion toward levator palpebrae are more important than bulk.
Procerus—Cosmetic	4 U at one procerus site WITHIN 20-U glabellar complex (not standalone) [55].	Controlled glabellar clinical evidence [8]; no independently established procerus trial dose.	Glabellar anatomical guidance [21]; no independent observational dose extracted.	2–4 U total as part of a glabellar-complex plan	Dose is driven by frown pattern and central brow depression rather than thickness.
Depressor supercilii—Cosmetic	No target-specific U.S. BOTOX Cosmetic aesthetic label dose established in the label checked [55].	No target-specific randomized/prospective dose established in the sources reviewed.	No separately quantified clinical case-study dose identified in the cited manuscript.	Low-dose micro-aliquot when separately targeted; generally incorporated within the glabellar-complex plan	No consistent starting range identified in the manuscript. Optional finesse target. Avoid diffusion toward levator palpebrae and excessive medial brow weakening.
Lateral orbicularis oculi—Cosmetic	24 U bilateral lateral canthal lines (12 U/side; 3 sites/side) [55].	Anatomical and clinical upper-face literature [9,40]; no separate study-arm dose extracted for this table.	Anatomical guidance [9]; no independently extracted observational dose.	4–12 U per side	Wrinkle extent and eyelid support dominate. Use lower dosing when lower-lid laxity or smile-asymmetry risk is present.
Medial orbicularis oculi—Cosmetic	No target-specific U.S. BOTOX Cosmetic aesthetic label dose established in the label checked [55].	No target-specific randomized/prospective dose established in the sources reviewed.	Anatomical considerations [47]; no clinical case-study dose independently extracted.	0.5–1 U micro-aliquots; approximately 1–2 U per side in selected cases	Very narrow safety window; consider dry eye, epiphora, diplopia, lacrimal proximity, lower-lid support, and asymmetry risk.
Nasalis/perinasal complex—Cosmetic	No target-specific U.S. BOTOX Cosmetic aesthetic label dose established in the label checked [55].	No target-specific randomized/prospective dose established in the sources reviewed.	Anatomical guidance and ultrasound studies [10,56]; not a clinical observational dose–response study.	Approximately 2 U per side for transverse nasalis as an anatomy-based starting point; broader patterns individualized	Treat the contributing muscle rather than the visible wrinkle alone; adjacent upper-lip function limits escalation.
Upper-lip elevator complex (LLSAN ± LLS/Zmi)—Cosmetic/functional smile modulation	No target-specific U.S. BOTOX Cosmetic aesthetic label dose established in the label checked [55].	Gummy-smile studies summarized in the existing systematic review [42]; individual randomized-study doses are not separately extracted from the manuscript.	Clinical and consensus discussions of smile-pattern-specific treatment [43,44].	2–4 U per side for most cases; 4–6 U per side in broader or mixed patterns	Smile pattern, gingival display and oral function guide interpretation; evidence does not establish a universal starting dose.
Orbicularis oris—Cosmetic/functional	No target-specific U.S. BOTOX Cosmetic aesthetic label dose established in the label checked [55].	No target-specific randomized/prospective dose established in the sources reviewed.	Clinical literature synthesized in a systematic review [45] and perioral technique review [46]; dose-specific case extraction not available.	Lip flip: 4–6 U total; perioral lines: ~1 U per point, usually 2–6 U total	Oral competence and function set the dosing ceiling; avoid aggressive escalation.
Depressor anguli oris (DAO)—Cosmetic/functional vector correction	No target-specific U.S. BOTOX Cosmetic aesthetic label dose established in the label checked [55].	No target-specific randomized/prospective dose established in the sources reviewed.	Anatomical study of DAO and DLI [23]; not a clinical observational dose–response study.	2–4 U per side	Avoid spread to DLI or orbicularis oris. Combination treatment may be preferable to increasing DAO dose.
Depressor labii inferioris (DLI)—Functional/asymmetry correction	No target-specific U.S. BOTOX Cosmetic aesthetic label dose established in the label checked [55].	No target-specific randomized/prospective dose established in the sources reviewed.	Three-dimensional anatomical investigation [23]; not a clinical observational dose–response study.	No standardized aesthetic starting dose established in the source manuscript; individualized assessment only.	No consistent starting range identified in the manuscript. Treat only when the responsible vector is clearly identified.
Mentalis—Cosmetic/functional	No target-specific U.S. BOTOX Cosmetic aesthetic label dose established in the label checked [55].	No target-specific randomized/prospective dose established in the sources reviewed.	Cadaveric/ultrasound anatomical study [24]; not a clinical observational dose–response study.	2–4 U per side	Depth and distribution are more important than bulk; thickness alone does not justify major dose escalation.
Platysma—Cosmetic/contouring	26, 31 or 36 U total for moderate-to-severe platysma bands, depending on band configuration [55].	Phase 3 randomized, placebo-controlled onabotulinumtoxinA study [37]; dose should be interpreted within the trial treatment pattern.	Anatomical and consensus literature [22,26,36]; no single observational dose independently extracted.	26–36 U total in the U.S.-labeled platysma-band protocol; individualization outside that protocol is not a validated starting range.	Safety depends on field and depth; avoid excessive weakening/spread that may affect neck function or swallowing.
Masseter—Contouring/functional masticatory	No target-specific U.S. BOTOX Cosmetic aesthetic label dose established in the label checked [55].	Randomized dose-finding study of a novel BoNT-A formulation [27]; randomized, triple-blind clinical trial [30]; prospective ultrasound-guided treatment study [16]. Study-specific formulations and denominators differ; no cross-product unit conversion is applied.	Clinical and anatomical literature on hypertrophy and tailored treatment [15,17,18,28,29].	20–40 U per side as an onabotulinumtoxinA-oriented practical range; individualize by thickness, volume, architecture, prior treatment, and product	The proposed 20–40 U/side is an onabotulinumtoxinA-oriented narrative synthesis, not an approved aesthetic dose. Reassess volume, function and prior-treatment atrophy.
Temporalis—Contouring/functional masticatory	No target-specific U.S. BOTOX Cosmetic aesthetic label dose established in the label checked [55].	Temporalis change following BoNT-A has been investigated [52]; a standardized aesthetic randomized dose was not identified in the cited manuscript.	Case-based evidence on unilateral/bilateral temporalis hypertrophy [49,50,51]; one case report describes 25 U of Botulax in one temporalis [50]. Product-specific units are not converted.	No standardized aesthetic dose; if treated, use case-based conservative dosing and reassess after prior masseter/temporalis treatment	Limited aesthetic-specific evidence; a single case report must not be treated as an established dose recommendation. Reassess muscle volume with repeated treatment.
Parotid gland—Glandular/contouring/secretory	No target-specific U.S. BOTOX Cosmetic aesthetic label dose established in the label checked [55].	No target-specific randomized/prospective dose established in the sources reviewed.	Anatomical/ultrasonographic study [32]; not a clinical observational dose–response study.	No standardized aesthetic starting dose established; indication-specific assessment and individualized planning. Ultrasound may assist localization in selected cases.	Glandular/secretory and aesthetic indications are not interchangeable. Do not infer an aesthetic approved dose from therapeutic sialorrhea labeling; consider dysphagia, xerostomia and adjacent-structure risk.
Submandibular gland—Glandular/contouring/secretory	No target-specific U.S. BOTOX Cosmetic aesthetic label dose established in the label checked [55].	No target-specific randomized/prospective dose established in the sources reviewed.	Anatomical/ultrasonographic study [31]; not a clinical observational dose–response study.	No standardized aesthetic starting dose established; indication-specific assessment and individualized planning. Ultrasound may assist localization in selected cases.	Glandular/secretory and aesthetic indications are not interchangeable. Do not infer an aesthetic approved dose from therapeutic sialorrhea labeling; consider dysphagia, xerostomia and adjacent-structure risk.

Notes. U = product-specific units; “per site”, “per side” and “total bilateral” are not interchangeable. U.S. regulatory labeling is formulation- and indication-specific. Absence of a separately extracted clinical-study dose does not establish absence of published evidence. Authors’ synthesis values derive from the source review and are not validated universal protocols. Clinical, functional, contouring and glandular indications must be interpreted separately. LLSAN = levator labii superioris alaeque nasi; LLS = levator labii superioris; Zmi = zygomaticus minor; DAO = depressor anguli oris; DLI = depressor labii inferioris.

### 2.5. Frontalis

The frontalis is the quintessential example of why facial BoNT-A dosing cannot be reduced to muscle size alone. It is a broad, thin, superficial elevator that inserts into the brow complex and forehead skin rather than functioning as a typical deeply anchored skeletal muscle [4,20,54]. Its aesthetic role is paradoxical: it causes horizontal forehead lines, yet it also keeps the brow elevated. Any dose sufficient to smooth the skin can also lower the brow if it is placed too low, too medially, too laterally, or too diffusely. For that reason, the frontalis is one of the few muscles in which a “smaller dose with better patterning” is often more elegant than a larger dose aimed at maximal paralysis [4,20,39,40].

The regulatory reference for onabotulinumtoxinA treatment of forehead lines is a total of 20 units distributed across five frontalis injection sites, administered together with 20 units for glabellar lines, yielding a combined treatment dose of 40 units [55]. This represents an approved regional treatment regimen rather than a universally appropriate dose for every frontalis muscle. Published anatomical reviews and expert recommendations describe individualized approaches that account for forehead dimensions, dynamic recruitment, baseline eyebrow position, and the need to preserve compensatory frontalis activity [4,20,40]. The 2025 Korean consensus describes lower forehead dose ranges for a different BoNT-A formulation [5]; these values should not be interpreted as directly interchangeable with onabotulinumtoxinA units [1,33]. Accordingly, Table 1 distinguishes the regulatory regimen from observational practice and the authors’ proposed synthesis. Any individualized starting range should be interpreted as a clinical proposal requiring anatomical assessment, rather than as a separately validated regulatory dose.

The best current evidence suggests that dose selection in the frontalis is driven by at least four factors. The first is forehead geometry: a tall, broad forehead may require more injection sites or slightly more total units simply to obtain even coverage. The second is movement pattern: some patients use the entire frontalis, while others recruit primarily the central or lateral fibers. The third is brow dependence: patients with heavy eyelids or subtle brow descent often overuse the frontalis as a compensatory elevator and over-treating them creates the classic “heavy forehead” complaint. The fourth is sex and muscle mass: men often have thicker and stronger frontalis muscles on ultrasound, but the dose increment that follows from this is usually modest rather than proportional [5,7,20,40,54].

The available anatomy and ultrasound literature reinforces a key principle: frontalis thickness is relevant but subordinate. Ultrasound measurements show that the frontalis is slightly thicker in men than in women and varies with body mass index (BMI) and laterality [7]. Cadaveric innervation studies show that the frontalis is not uniformly innervated, and recent work has proposed injection guidance based on the intramuscular innervation pattern of the temporal branch of the facial nerve [54]. These findings support individualized distribution of small aliquots along active fibers rather than blanket escalation of units. If the goal is natural rejuvenation, thickness should modify the dose in small steps; it should not justify abandoning conservative placement rules.

For practical interpretation, the labeled 20-unit frontalis regimen should remain the principal regulatory reference for onabotulinumtoxinA, while lower or otherwise individualized dosing approaches should be explicitly classified according to their supporting evidence [4,20,40,55]. The authors’ proposed starting range in Table 1 is intended to guide clinical reasoning rather than establish an independently validated dose–response threshold. Selection within any proposed range should consider forehead height, baseline brow support, compensatory frontalis recruitment, wrinkle distribution, and the patient’s preference for preserved movement. Particular caution is warranted in patients with pre-existing brow descent or limited brow-elevation reserve, because excessive weakening may produce brow ptosis, eyelid heaviness, or an unnatural expression [20,39,40]. Thus, the objective is not maximal muscular suppression but an individualized balance between wrinkle reduction and preservation of brow function.

In relation to the broader themes of this review, the frontalis teaches two lessons. First, dose cannot be separated from muscle function. Second, distributed injections are helpful only when they mirror the real vector of frontalis activity. Broad superficial distribution can reduce lines, but indiscriminate diffusion can flatten expression and worsen brow position. For this muscle, NMJ-targeted logic does not mean high-density puncture everywhere; it means selecting the active motor field and preserving the fibers the patient still needs for a balanced brow.

### 2.6. Corrugator Supercilii, Procerus, and Depressor Supercilii

The glabellar complex is among the most extensively studied regions in aesthetic BoNT-A treatment and provides an important example of the distinction between regional and muscle-specific dosing. The approved onabotulinumtoxinA regimen for glabellar lines comprises 20 units distributed across five injection sites: two sites in each corrugator supercilii and one in the procerus [8,55]. This five-site regimen is supported by clinical trial evidence and regulatory labeling, but the total regional dose should not be interpreted as an independently validated dose requirement for each contributing muscle. Anatomically, glabellar expression involves the corrugator supercilii and procerus, with variable contributions from the depressor supercilii and adjacent orbicularis oculi fibers [4,21]. Therefore, Table 1 separates the approved regional regimen from muscle-specific anatomical recommendations, clinical observations, and the authors’ proposed individualized approach. This distinction is particularly important when discussing lower-dose precision techniques that have not undergone the same level of clinical validation as the approved regional protocol.

The corrugator supercilii is deeper, more laterally extended, and more directly linked to vertical glabellar lines than the procerus [4,21]. It also lies in a region where depth matters enormously: excessively deep or low lateral injection can increase the risk of eyelid ptosis by allowing toxin to track toward the levator palpebrae superioris, while excessively high or medial placement can produce a “samurai eyebrow” or quizzical brow through frontalis imbalance [21,39]. The 2022 anatomical review by Yi and colleagues explicitly emphasized that the best injection points are determined by anatomy rather than by the eyebrow itself, particularly in older patients with brow descent [21]. In that anatomy-based review, the authors proposed a minimal corrugator-focused protocol of 6 units total, delivered as 1.5 units into each of four medial and lateral corrugator points, with the lateral point kept superficial and the medial point placed after contacting periosteum and withdrawing slightly [21]. This is substantially lower than the corrugator share of the labeled glabellar pattern, and its importance is conceptual: accurate anatomical targeting may reduce the dose required for some individuals.

In clinical practice, treatment planning commonly addresses the glabellar depressor complex rather than an isolated corrugator muscle. The approved 20-unit onabotulinumtoxinA regimen provides a standardized reference, whereas alternative dose distributions described in anatomical publications and expert recommendations reflect different degrees of individualization [4,8,21,55]. These approaches should not be considered equivalent in evidentiary strength. In particular, anatomy-guided low-dose recommendations may provide a rationale for conservative treatment in selected patients, but they do not establish clinical equivalence to the approved five-site regimen. When discussing individualized doses, the present review therefore emphasizes the underlying evidence category, the anatomical target, and the functional consequences of treatment rather than presenting a single universal per-muscle dose.

The role of thickness in the glabellar complex is nuanced. Ultrasound studies show that the corrugator is usually thicker than the frontalis and exhibits variability across sex and individual facial morphology [7]. Men and stronger frowners commonly require higher doses clinically, but the evidence does not demonstrate a simple linear correction where a 10% thicker corrugator demands a 10% higher dose. Rather, thicker or stronger corrugators justify movement toward the upper end of the usual range, especially when vertical lines are severe and the brow depressor pattern is broad [5,7]. In smaller or lower-BMI faces, or in patients at higher risk of ptosis, a conservative starting dose remains preferable, with touch-up if necessary. The glabella therefore supports a “thickness-modified but anatomy-limited” dosing model.

Ultrasound-guided evidence strengthens this interpretation. A prospective randomized 2024 study comparing real-time ultrasound-guided injection with conventional blind glabellar injection found faster onset and greater wrinkle-score reduction in the ultrasound-guided group [35]. This finding is clinically important because it suggests that at least part of variability in glabellar response may be technical rather than biological. If the corrugator belly, procerus depth, and brow depressor vectors are accurately identified, the injector may achieve more reliable effect without automatically increasing units. Although the study was not primarily designed as a pure unit-sparing trial, its results are consistent with the broader endplate-targeting literature [11,12,13,35].

For the procerus specifically, the literature supports a modest dose in a deep midline injection, often around 2 to 4 onabotulinumtoxinA units when treated as an individual muscle, although in standardized glabellar patterns it frequently receives 4 units as part of the five-point scheme [8,21,33]. The procerus is less often the determinant of total regional dose than the corrugator, but it is often the determinant of whether the patient’s central horizontal glabellar contraction fully relaxes. Overemphasis on corrugator dosing with under-treatment of the procerus can leave residual central activity; the converse can flatten the central brow without adequately addressing vertical frown lines.

Taken together, the glabellar literature supports a layered conclusion. Standard on-label dosing remains highly defensible for reproducible treatment of the region. However, anatomy-driven approaches suggest that specific muscular components, especially the corrugator, may respond to lower doses when injection depth and point selection are optimized [8,21,33]. Thickness and sex modify the dose, but they do not replace the need to understand the shape of the brow depressor complex. For the glabella, precision is not a luxury; it is part of the dose.

The depressor supercilii merits separate mention because it is frequently hidden inside the glabellar dose rather than named as an independent target. It draws medial brow skin inferomedially and helps deepen vertical glabellar lines and medial brow descent. Vertical glabellar lines reflect combined corrugator, depressor-supercilii, and procerus vectors rather than corrugator action alone [21]. When a visibly active depressor-supercilii vector remains after standard corrugator/procerus treatment, a very small supplemental dose may be used, but it should be treated as a finesse adjustment rather than as a routine high-dose muscle.

When the depressor supercilii is intentionally treated, conservative microdosing within the total glabellar-complex plan is preferable to routine additional dosing. The injection should remain high enough above the orbital rim and precise enough to avoid diffusion toward the levator palpebrae superioris. Thickness has little independent value here; the relevant dosing variables are brow-depressor vector, medial brow position, ptosis risk, and whether the patient already relies on frontalis recruitment.

### 2.7. Lateral and Medial Orbicularis Oculi

Lateral canthal lines are a well-established aesthetic indication for onabotulinumtoxinA, but the evidence must be distinguished from anatomy-based recommendations for individualized treatment. The U.S. BOTOX Cosmetic prescribing information specifies 24 units for bilateral lateral canthal lines, administered as 12 units per side across three injection sites [55]. This is an approved regional treatment regimen, not a universal dose requirement for every orbicularis oculi presentation. Anatomical studies and expert recommendations describe alternative distributions intended to account for muscle activity, wrinkle extension, and proximity to functionally sensitive periocular structures [4,9,40]. Such individualized approaches should be identified as anatomical or expert-derived recommendations rather than assumed to have the same evidentiary status as the labeled regimen. Table 1 therefore reports the regulatory dose separately from the authors’ proposed clinical synthesis.

Rather than treating these recommendations as contradictory, it is more useful to recognize that they address different clinical philosophies. The labeled dose is a standardized efficacy program intended to work across a wide range of patients with moderate to severe lateral canthal lines. The anatomical review is an efficiency- and safety-oriented proposal that prioritizes minimally effective dosing at the border of the orbicularis oculi muscle [9,33]. Both imply that the orbicularis oculi are best treated with multiple superficial points near the active edge of the muscle rather than with a single large bolus. The key point is that the lateral canthus is a region where coverage and safety must be balanced carefully.

Thickness contributes less to dose selection here than pattern and surrounding risk. Ultrasound observations suggest that the lateral orbicularis can be the thickest part of the muscle around the eye, but the absolute thickness remains small compared with the masseter or even the corrugator [7,9]. The practical dose question is therefore not “How thick is the muscle?” but “How far do the rhytids extend, how strong is the squeeze, what is the patient’s eyelid support, and what is the risk of unwanted spread?” Patients with lower-lid laxity, prior blepharoplasty, dry eye tendency, or preexisting smile asymmetry often benefit from the lower end of the dosing range and from particularly careful superior and inferior placement [4,9,38,39].

For lateral orbicularis oculi treatment, the approved 12-unit-per-side onabotulinumtoxinA regimen provides the clearest regulatory reference [55]. Lower-dose approaches discussed in anatomical and expert literature may be considered in selected patients, but the evidence does not establish that a particular reduced dose is universally equivalent to the labeled regimen [9,40]. The proposed 4–12-unit-per-side range in Table 1 should therefore be interpreted as an authors’ synthesis encompassing conservative individualized approaches and the labeled dose, rather than as a dose range validated in a single comparative trial. Treatment planning should prioritize the distribution of lateral canthal rhytids, contraction strength, baseline eyelid function, and the risk of unintended weakness. Dose escalation should not be based on muscle thickness alone, particularly in this thin, functionally important periocular muscle.

The orbicularis oculi also illustrate a broader principle about distributed injection. In this muscle, distributed superficial aliquots do not merely enhance coverage; they actively reduce risk by allowing the injector to stay outside deeper, more dangerous planes. If one wants a practical example of how “more precise distribution may save units,” the lateral canthus is a good candidate. A carefully chosen three-point, lower-dose pattern may be sufficient in many patients, whereas a broader standardized protocol may be reserved for higher-severity cases or for injectors following a trial-validated regimen [9,33]. The dose, again, is inseparable from the map.

The medial wrinkle pattern of the orbicularis oculi muscle should be distinguished from classic lateral canthal rhytids. Medial eye wrinkles are often formed by a small medial preseptal or pretarsal orbicularis component and by interaction with adjacent nasal-root and glabellar vectors. The published anatomical guideline for medial eye wrinkles supports conservative, superficial microdosing because the region is functionally sensitive and adjacent to the lacrimal apparatus, extraocular structures, nasal sidewall, and medial canthal tendon [47].

Medial orbicularis oculi treatment should be considered separately from the approved lateral canthal-line indication. The anatomical guidance described by Yi and Wan supports a conservative approach to focal medial eye wrinkles, but does not establish a regulatory dosing standard or a broadly validated dose–response relationship [47]. The micro-aliquot approach summarized in Table 1 (approximately 0.5–1 unit per site, or 1–2 units per side) is therefore presented as an anatomy-informed authors’ synthesis, not as an approved or universally validated regimen. Because the medial periocular region contributes to eyelid closure and tear-film function, treatment decisions should account for baseline eyelid support, ocular symptoms, and the possibility of dry eye, epiphora, asymmetry, or unintended weakness. These functional considerations are more important than muscle thickness as an isolated dosing determinant.

### 2.8. Nasalis and Dynamic Nasal Wrinkles

Dynamic nasal wrinkles arise from more than one muscular pattern. Cadaveric dissection and dynamic ultrasonography have demonstrated a muscle-free “bunny triangle” beneath the visible bunny-line region, bordered by the procerus, nasalis, orbicularis oculi, and levator labii superioris alaeque nasi (LLSAN) [56]. Thus, the visible wrinkle should not be assumed to directly overlie a single target muscle. The transverse part of the nasalis contributes to nasal sidewall scrunch lines, whereas oblique bunny lines may involve the LLSAN and medial orbicularis oculi in addition to the nasalis [10,56]. The therapeutic window is narrow: insufficient dose leaves residual scrunching, but excessive or misplaced toxin may alter upper-lip animation, nasal smile, or the patient’s expressive balance. Anatomical literature therefore favors small, superficial doses placed according to the observed contraction pattern rather than broad empiric escalation [10,56].

The 2022 anatomical review of the nose region provides muscle-specific guidance for these patterns. For nasal sidewall scrunch lines produced by the transverse nasalis, 2 units are recommended at the superior ala on each side. For oblique bunny lines, the same review recommends 2 units per side directed to the upper LLSAN at an anatomically defined point, reflecting the contribution of adjacent perinasal muscles rather than treating all nasal wrinkles as a single nasalis indication [10]. A 2025 Korean consensus similarly recommends 2 units per side for the transverse nasalis component of dynamic nasal wrinkles [5].

The literature on dynamic nasal wrinkles is valuable because it highlights the difference between local wrinkle treatment and muscle-complex treatment. Dynamic ultrasonography has confirmed nasalis thickening during bunny-line formation in most examined volunteers, while also demonstrating that the visible wrinkle may overlie the muscle-free bunny triangle [56]. Accordingly, when transverse nasalis contraction is dominant, a low dose directed to the nasalis is usually sufficient. When oblique bunny lines reflect stronger LLSAN or adjacent perinasal activity, the target should be adjusted to the responsible vector rather than simply increasing the nasalis dose [5,10,56]. Thickness has only a minor role here. The relevant variables are depth, contraction pattern, and whether the visible wrinkle maps to nasalis activity or to adjacent perinasal recruitment.

For dynamic nasal wrinkles, approximately 2 units per side is described in anatomy-based recommendations for selected transverse nasalis patterns [10]. This value should be interpreted as a published anatomical recommendation rather than a regulatory-label dose or a prospectively validated universal starting dose. The Korean consensus also discusses a 2-unit-per-side approach, but formulation-specific dosing must be considered before applying numerical recommendations across BoNT-A products [1,5]. Importantly, bunny lines may arise from nasalis activity, adjacent perinasal muscles, or combined contraction patterns, and the visible wrinkle does not necessarily overlie the responsible muscle [10,56]. Accordingly, Table 1 presents the nasalis dose as an authors’ synthesis informed by anatomical literature. Individualized assessment of contraction pattern and adjacent functional structures should take precedence over automatic dose escalation or reliance on muscle thickness alone.

### 2.9. Upper-Lip Elevator Complex for Gummy Smile

The dosing literature for gummy smile differs from most BoNT-A literature because the clinically visible problem is not a single wrinkle or a single named muscle. Rather, excessive gingival display may reflect overactivity of one or more upper-lip elevator muscles, most commonly the levator labii superioris alaeque nasi (LLSAN), levator labii superioris, zygomaticus minor, and in mixed patterns the zygomaticus major or other accessory elevators [6,42,43,44]. As a result, the literature rarely presents a simple per-muscle dose in the same way that it does for the corrugator or masseter. Instead, most publications discuss injection patterns such as the Yonsei point, the LLSAN point, or anterior, posterior, and mixed gummy-smile patterns, each of which represents an attempt to weaken a functional muscle ensemble rather than an isolated muscle belly [42,43,44].

The 2024 systematic review and bibliometric analysis of gummy smile management by Wang and colleagues confirms this heterogeneity. Across 13 prospective controlled clinical trials, dose and injection strategy varied according to gingival exposure and the presumed muscular pattern. The review concluded that a small dose of approximately 2 to 3 units can be adequate for mild gummy smile, while escalation to 5 units or higher may be considered when greater correction is required, with a corresponding increase in adverse-effect risk [42]. The LLSAN emerged as the principal target for anterior and mixed-type gummy smile [42]. This evidence supports severity- and pattern-based titration rather than a single fixed dose for all patients.

A smaller but very pragmatic perspective was offered by Dimasi and colleagues in 2023, who argued that mild-to-moderate gummy smile can often be treated effectively with 4 units per side at the Yonsei point, thereby simplifying what had sometimes become an overly complicated pattern taxonomy [43]. Their approach is appealing because the Yonsei point seeks to influence the convergence zone of the LLSAN, levator labii superioris, and zygomaticus minor. From a dosing perspective this is an example of motor-zone logic: if one well-chosen point can intercept the action of several upper-lip elevators, the injector may be able to use a modest amount of toxin and still obtain a visible reduction in gingival display [42,43]. This does not mean that all gummy smiles should be treated with a single point, but it does support the broader principle that anatomically efficient placement may substitute for indiscriminate dose escalation.

Consensus literature from the Middle East and other expert groups supports a similar range. These sources generally recommend 2 to 4 units per side for straightforward anterior gummy smile and suggest a practical heuristic of approximately 1 unit per millimeter of gingival exposure up to a moderate ceiling, with more complex mixed patterns treated by adding an additional point or broadening the target to include the zygomaticus complex [44]. Such recommendations are clinically useful because they translate the visible severity of the smile into a small, bounded range rather than encouraging the injector to pursue complete upper-lip paralysis. The upper lip is highly expressive and unforgiving; over-treatment can alter phonation, eating, lip seal, and smiling identity.

Gummy-smile treatment illustrates why muscle thickness alone cannot determine BoNT-A dose. Although the upper-lip elevator muscles are relatively small, limited weakening may produce a clinically visible change in gingival display because treatment modifies a coordinated and functionally important movement pattern [6,42,43,44]. Published studies and expert recommendations describe heterogeneous doses, injection sites, formulations, and gummy-smile classifications. Consequently, the 2–4-unit-per-side range and the broader 4–6-unit-per-side approach summarized in Table 1 should be interpreted as an authors’ synthesis of selected clinical recommendations, not as a single dose range established by pooled comparative evidence. Treatment selection should be guided by the pattern and severity of gingival exposure, the muscles contributing to lip elevation, and preservation of natural smiling function. The risk of upper-lip weakness, asymmetry, altered articulation, and impaired oral competence warrants conservative interpretation of all proposed dose ranges.

This muscle group also illustrates the importance of formulation awareness. Some reviews report doses using abobotulinumtoxinA or other formulations, and the absolute unit numbers cannot simply be transplanted into onabotulinumtoxinA-based practice [1]. For a review focused on practical facial-muscle dosing, the safest summary is therefore to describe the upper-lip elevator complex as a low-dose precision target in which point selection is more important than a large total number of units. Distributed injection can help if the smile pattern is broad, but the distribution should be driven by the smile vector and the muscle ensemble rather than by an attempt to “cover” the whole upper lip.

### 2.10. Orbicularis Oris for Lip Flip and Perioral Lines

Orbicularis oris presents one of the narrowest therapeutic windows in facial BoNT-A practice. Unlike the frontalis or masseter, which tolerate a moderate range of total units if the pattern is correct, the perioral sphincter can quickly become dysfunctional if dose is excessive or poorly distributed. Speech articulation, straw use, whistling, kissing, lip seal, and oral competence all depend on a finely balanced orbicularis function [6,45,46]. For that reason, the orbicularis oris is best understood as a finesse target rather than a “dose-driven” target. Small doses are the rule, and they are preferably divided into multiple superficial micro-aliquots.

Contemporary literature divides orbicularis oris treatment into two related but distinct indications. The first is the so-called lip flip, in which tiny amounts of toxin are placed into the superficial upper orbicularis to reduce inward rolling and allow slight eversion of the vermilion. The second is treatment of perioral rhytids, in which superficial doses are placed around the lip to soften smoker’s lines or barcode lines [45,46]. The 2025 systematic review of the lip flip concluded that the procedure typically uses 4 to 6 units of BoNT-A in total, most often placed into four points of the upper orbicularis oris, and that patient satisfaction is generally good when careful selection and counseling are used [45]. This is a useful anchor because it confirms that a total in the low single digits can be clinically meaningful in the perioral region.

Technique-oriented reviews reinforce the same dosage philosophy. Smit’s 2024 chapter on perioral enhancement described superficial injections of approximately 1 unit of onabotulinumtoxinA per point for perioral lines, along with similarly conservative dosing for lip eversion procedures [46]. The narrative review of oral and maxillofacial BoNT techniques likewise places perioral treatment firmly in the “micro-dose” category, emphasizing symmetry, superficial placement, and staged retreatment rather than front-loaded unit escalation [6]. The consistency across these sources is striking; although exact patterns differ, most authors converge on 2 to 6 total units for upper-lip flip indications and a similarly low-per-point philosophy for perioral lines.

From a muscle-specific standpoint, the orbicularis oris also demonstrates why thickness is a poor standalone guide. The muscle may be palpably stronger or thicker in some patients, especially in habitual puckering or smoking-related patterns, but the relevant determinant is not simply bulk. It is the functional consequence of partial denervation in a sphincter. Even if a thicker orbicularis oris might theoretically tolerate a slightly larger dose, the clinician is constrained by speech and competence risk long before muscle thickness becomes the dominant variable [6,45,46]. As a result, practical dose adjustment is usually performed by changing the number of points or by adding a small incremental unit at a later visit rather than by doubling the initial dose.

The dose literature for orbicularis oris should be interpreted separately for upper-lip eversion and perioral rhytid treatment. For a lip flip, the published procedural literature describes small total doses, commonly around 4–6 units distributed across several superficial injection points [45]. For perioral rhytids, technique-oriented recommendations describe approximately 1 unit per site, with total dose varying according to the distribution of lines and the number of treated sites [6,46]. These values are presented in Table 1 as an authors’ synthesis of procedural literature, not as U.S. regulatory-label doses or universally validated treatment protocols. Formulation-specific units, treatment objectives, and the distinction between per-site and total dose must be preserved when comparing reports [1,33]. Because orbicularis oris is essential for articulation, lip seal, drinking, and other oral functions, conservative individualized dosing and avoidance of unintended sphincter weakness are more important than increasing dose according to muscle bulk alone.

The broader lesson is that the orbicularis oris behaves like the frontalis in one respect and unlike the masseter in another. Like the frontalis, it is functionally essential and sensitive to small dose differences. Unlike the masseter, it is not treated for bulk reduction. Therefore, thickness should not be used to justify substantially higher units. Precision, symmetry, and staged treatment matter far more than muscle size.

### 2.11. Depressor Anguli Oris and Depressor Labii Inferioris

The depressor anguli oris (DAO) is one of the most frequently treated lower-face muscles because excessive downward pull at the oral commissure contributes to a sad or fatigued expression. Yet the DAO is also one of the easiest muscles to over-treat. It lies in a crowded functional neighborhood that includes the depressor labii inferioris (DLI), platysma, risorius, and portions of the orbicularis oris, and diffusion into any of these structures can alter smile symmetry or lower-lip control [6,22,23,25]. Accordingly, DAO dosing in the literature is characteristically modest. The recurring theme is not “how many units can be used?” but “how few units can achieve the desired corner elevation without collateral weakness?”

For depressor anguli oris (DAO) treatment, anatomical studies, procedural reviews, and expert recommendations generally favor conservative, anatomically targeted injection because of the close functional relationship between the DAO, depressor labii inferioris (DLI), and adjacent perioral muscles [6,22,23,25]. However, these sources should not be interpreted as establishing a universally validated dose–response relationship. DAO treatment remains an off-label aesthetic application, and the strength of evidence supporting a particular dose is lower than that available for several approved upper-face indications. Accordingly, the proposed DAO dose in Table 1 is classified as an authors’ synthesis of published clinical recommendations rather than as a regulatory or prospectively validated standard. Treatment planning should prioritize the direction of oral commissure movement, baseline smile symmetry, and avoidance of unintended lower-lip weakness.

The DLI is a different story. It is less commonly injected deliberately, and in the aesthetic literature it is more often discussed as a structure to be avoided than as a primary target [23]. Selected lower-face asymmetry patterns or lower-lip eversion problems may justify treating the DLI directly, but the evidence base is thin, and dosing recommendations are therefore conservative. Three-dimensional lower-face analyses suggest that the DLI should be approached only when the clinician is confident in distinguishing its vector from DAO-driven mouth-corner depression [23]. In practical terms, when the DLI is treated directly, very low, conservative dosing is preferable to broader suppression [23,25].

The dose logic of the DAO again illustrates why facial BoNT-A cannot be standardized solely by muscle thickness. The DAO is relatively thin and superficial, yet it can exert a strong visible effect because of its leverage on the oral commissure. As with other thin perioral muscles, high doses may be counterproductive because neighboring muscles are critical for natural smile mechanics [23,25]. Therefore, the best evidence-based direction is to think in terms of small superficial aliquots and functional vector correction rather than broad denervation.

Several practical conclusions can be drawn from the available DAO and DLI literature. First, the proposed 2–4-unit-per-side onabotulinumtoxinA-oriented range for DAO treatment in Table 1 should be understood as an expert- and anatomy-informed synthesis rather than a dose established through adequately powered comparative trials [6,22,25]. Second, deliberate DLI treatment requires a distinct functional assessment and should not be assigned the same dosing recommendation as the DAO merely because the muscles are adjacent [23,25]. No universally established aesthetic starting dose for the DLI can be inferred from the evidence summarized here. Third, lower-face treatment outcomes depend on the interaction of several muscular vectors; therefore, an unsatisfactory result should not automatically prompt DAO dose escalation. The risks of asymmetric smiling, impaired lower-lip movement, and altered oral competence warrant particular caution when interpreting these off-label dosing recommendations.

The lower-face lift literature adds another interesting perspective. Lorenc and colleagues argued that creating lift in the lower face with botulinum toxin A often depends on coordinated treatment of several depressor vectors rather than maximal weakening of a single muscle [25]. This concept is important for dose-focused review because it explains why the DAO can remain a low-dose muscle even in patients with conspicuous downturned corners: the injector can improve outcome by treating adjacent contributors rather than by escalating the DAO units beyond the safe and anatomically conservative range.

### 2.12. Mentalis

Mentalis is a compact but highly expressive midline lower-face muscle whose overactivity produces chin dimpling, soft-tissue bunching, and in some patients a shortened or strained lower-face appearance. Recent anatomical work makes it a good example of how modern anatomy can refine both dose and depth [24]. The literature supports anatomically deliberate targeting that accounts for the muscle’s layered architecture and its relationship to the overlying soft tissue.

Cadaveric and ultrasonographic work by Choi and colleagues identified effective injection locations based on the morphology and distribution of the mentalis and emphasized that its upper and lower portions do not behave identically [24]. These findings support selecting injection depth and location according to the target anatomy rather than relying on a superficial uniform injection pattern.

From a dose-review perspective, the mentalis illustrates the distinction between anatomical precision and evidence-based dose determination. Published anatomical and procedural sources support individualized targeting according to muscle morphology, contraction pattern, and the relationship between the mentalis and lower-lip function [6,24,25]. However, anatomical localization studies do not independently establish an optimal clinical dose. The low single-digit doses discussed in the procedural literature, including the approximately 2–4-unit-per-side range summarized in Table 1, should therefore be classified as an authors’ synthesis rather than a prospectively validated universal starting regimen. The available evidence does not justify increasing dose solely on the basis of muscle thickness, particularly when the treatment objective is reduction in chin dimpling rather than broad muscular weakening.

Direct evidence linking mentalis thickness to dose is limited. Morphology may influence placement and depth, but dose selection is driven primarily by the severity of chin dimpling, the degree of mentalis strain at rest and animation, the presence of lower-lip incompetence, and whether adjacent lower-face muscles are also being treated [6,24,25]. Thus, muscle thickness should not be used as a stand-alone reason for major dose escalation.

Mentalis also demonstrates the clinical value of correct depth in relation to unit economy. Because the muscle sits over bone and contributes to both projection and dermal pebbling, injection depth should be selected according to the intended target and contraction pattern. Anatomically distributed injections can therefore improve treatment precision without necessarily increasing total dose [24].

In practical onabotulinumtoxinA-oriented terms, the approximately 2–4-unit-per-side range shown in Table 1 represents a conservative synthesis of procedural recommendations, not a dose independently established by a comparative clinical trial [6,24,25]. Injection distribution and depth should be individualized according to mentalis contraction and anatomical relationships. Particular attention is required in patients with pre-existing lower-lip incompetence or asymmetry, because excessive weakening may alter lip posture, oral competence, and facial expression. The limited direct dose–response evidence favors cautious interpretation of proposed ranges rather than routine escalation based on apparent muscle bulk.

### 2.13. Platysma

Platysma occupies a special place in facial BoNT-A dosing because it is simultaneously thin, extensive, and functionally important. Its treatment spans two related indications: platysmal band reduction and lower-face or jawline lifting. Unlike the masseter, the platysma is not thick in absolute terms, yet total dose can still be substantial because the treatment field is large and often bilateral [22,25,26,36,37]. This feature makes platysma dosing particularly informative in a dose-focused review. It reminds us that total dose is not simply a function of muscle thickness; it is also a function of surface area, vector distribution, and the number of active bands that require treatment.

Historically, lower-face and neck consensus statements described platysma treatment in broad practical terms, often recommending about 2 units per point at several points along each visible band, with total bilateral doses around 40 units or more depending on the extent of banding [22,26]. These early frameworks remain recognizable in modern practice because they match the visible anatomy of platysmal bands: each band is a long contractile strip rather than a compact focal muscle belly. However, more recent work has introduced a far more nuanced understanding of where along the muscle toxin may be most useful.

The 2022 platysma review by Yi and colleagues emphasized anatomy-based targeting for both platysmal bands and jawline lifting, again favoring approximately 2 units per point but stressing that the treatment points should follow the actual distribution of active fibers and visible bands rather than an arbitrary grid [26]. This laid the groundwork for the 2025 motor-nerve distribution paper, which specifically proposed that injections guided by the distribution of motor innervation may achieve effective weakening with fewer sites than conventional blanket treatment [36]. In other words, the platysma may be an important facial example of the principle that NMJ- or motor-zone-aware placement can reduce injection burden and potentially improve efficiency.

The strongest clinical evidence discussed in this review comes from the phase 3 randomized trial evaluating onabotulinumtoxinA for platysma prominence [37]. The study assessed severity-based total doses of 26, 31, or 36 units, providing prospective evidence for a defined treatment protocol rather than for unrestricted dosing across all platysma-related aesthetic indications. These dose levels are also reflected in the U.S. BOTOX Cosmetic prescribing information for platysma bands [55]. Importantly, this evidence should not be extrapolated without qualification to isolated jawline-lifting procedures, alternative injection distributions, or other BoNT-A formulations. The treatment field, distribution of visible bands, and functional anatomy are relevant to dose selection, but the available trial does not establish muscle thickness as an independent predictor of optimal platysma dose. Table 1 therefore reports the regulatory regimen separately from historical consensus-based and anatomy-guided approaches.

How, then, should muscle thickness be understood in the platysma? Strictly speaking, it is a poor primary determinant because the platysma is thin in most patients regardless of whether the neck bands are severe. What changes is not thickness so much as field size, band prominence, insertion pattern, and the degree to which the platysma contributes to jawline depression versus isolated neck banding [26,36,37]. Therefore, platysma dosing behaves more like area-based or vector-based dosing than thickness-based dosing. A larger, stronger, more extensive platysmal pattern deserves a higher total dose, but not because the muscle is thick in the same sense as the masseter.

Platysma literature also offers one of the clearest links between distributed injections and potential unit economy. When toxins are deposited in many small units along the visibly active bands or the upper jawline platysma, the clinician can create a smooth, distributed effect without forcing a large amount of toxin into a small number of points. More importantly, the motor-nerve distribution study suggests that not all traditionally used points are equally valuable [36]. If future clinical trials confirm that targeted sites outperform generic grids, platysma treatment may become more efficient in both site number and total unit exposure.

For practical interpretation, two distinct evidence categories should be maintained. Historical consensus statements and anatomical reviews describe distributed treatment of visible platysmal bands, including approximately 2 units per injection point, with total dose varying according to the number and extent of treated bands [22,26]. These approaches represent expert- and anatomy-informed practice and should not be presented as equivalent to a validated regulatory protocol. By contrast, the 26-, 31-, and 36-unit onabotulinumtoxinA regimens evaluated in the phase 3 study provide a stronger evidence base for the defined platysma-band treatment indication [37,55]. The authors’ synthesis in Table 1 prioritizes this established regimen while acknowledging that other platysma-related techniques have different objectives and less standardized supporting evidence. Accordingly, dose selection should not be based on muscle thickness alone or on an assumed equivalence between neck-band reduction and jawline lifting.

Platysma treatment requires particular attention to regional safety because the muscle is anatomically extensive and closely related to structures involved in lower-face and neck function. Excessive or unintended weakening may contribute to dysphagia, neck weakness, altered lower-face movement, or facial asymmetry [22,26,37,55]. Consequently, treatment should not be justified by the assumption that a greater number of injection sites or a higher total dose necessarily improves outcomes. The approved onabotulinumtoxinA regimen provides a defined reference for platysma bands, whereas alternative anatomy-guided approaches require separate interpretation of their benefits, limitations, and safety evidence. This distinction is essential when translating findings from randomized trials into individualized clinical practice.

### 2.14. Masseter and Temporalis

Among facial muscles, the masseter provides the clearest and most clinically relevant evidence that dose correlates with muscle size and thickness. This is partly because the treatment goal is often not only movement reduction but also visible volume reduction. In contrast with the frontalis, DAO, or orbicularis oris, the masseter is frequently treated as a true contouring muscle. The aesthetic objective is to reduce lower-face width or soften a hypertrophic mandibular angle, and success can be measured not only by patient impression but also by ultrasound thickness, three-dimensional contour change, or facial width metrics [15,16,17,18,27,28,29,30]. For that reason, the masseter occupies a special place in a dose-focused review of facial BoNT-A dosing: it is the muscle in which the logic of dose, thickness, and injection architecture becomes most explicit.

The contemporary literature consistently shows that masseter treatment requires larger doses than most other facial targets. A 2025 comprehensive review by Ferrillo and colleagues summarized a broad literature base and noted that common recommendations for onabotulinumtoxinA- or incobotulinumtoxinA-oriented practice often fall between approximately 24–60 units total within the treatment zone, typically divided among three deep injection points in the lower half of the muscle [15]. Narrative reviews focused on oral and maxillofacial aesthetics similarly cite approximately 25 to 30 units per side as a frequent practical starting point in routine contouring practice [6]. Lower-dose approaches exist, especially in mild prominence or for patients seeking only modest reduction, but they are generally the exception rather than the rule.

Randomized dose-finding evidence provides support for a dose-dependent contouring response in the masseter, although the findings remain formulation-specific. Hong and colleagues evaluated prabotulinumtoxinA in a randomized, double-blind, placebo-controlled study of benign masseteric hypertrophy and reported greater treatment effects with selected higher-dose regimens than with the lowest tested regimen [27]. Because the study used prabotulinumtoxinA, its numerical dose levels cannot be directly converted into onabotulinumtoxinA recommendations [1,33]. Nevertheless, the study supports the importance of considering baseline muscle prominence, treatment objectives, and objectively measured changes in muscle thickness when evaluating contouring outcomes. It does not establish a universal linear relationship between baseline thickness and optimal dose, nor does it justify automatic dose escalation in every patient with a relatively large masseter.

Personalized treatment studies deepen this point. Xie and colleagues classified masseter hypertrophy patterns and adjusted both total dose and number of injections points accordingly, using approximately 20 to 40 units per muscle with one to three sites depending on the bulge pattern [29]. Their tailored protocol reduced mean sonographic thickness from 12.9 ± 2.9 mm to 8.7 ± 1.7 mm at three months and achieved high patient satisfaction. This study is particularly valuable because it moves beyond generic dose ranges and shows how muscle architecture and thickness can directly shape a customized plan. A thick, broad, compartmentalized masseter may justify not only more units but also more thoughtful distribution across the active lower half of the muscle.

Longitudinal observations further indicate that the masseter changes over the course of treatment. Kim and colleagues reported associations between baseline muscle thickness, treatment-related thickness reduction, and changes observed during repeated treatment [28]. These findings suggest that the anatomical characteristics used to guide an initial injection may not remain constant at subsequent visits. Accordingly, reassessment of muscle prominence, contraction strength, facial contour, treatment history, and functional symptoms is appropriate before selecting a repeat dose. A reduction in muscle thickness may support reconsideration of the original regimen, but the available evidence does not establish a validated maintenance-dose reduction formula. Repeating an initial dose without reassessing the current target anatomy may therefore be less appropriate than an individualized approach based on the patient’s present clinical findings.

The masseter literature also demonstrates why injection architecture matters as much as total units. Ultrasonographic guidance and architectural analyses show that the masseter is not a uniform block. The deep inferior tendon can partition the muscle and restrict toxin spread, while regional thickness differences may be concealed by overlying soft tissue [17,18]. Lee and colleagues emphasized ultrasonographic considerations for safe and efficient injections, and Shi and colleagues showed that deep inferior tendon morphology has practical implications for how evenly toxin can distribute within the muscle [17,18]. These findings help explain paradoxical bulging after apparently adequate dosing: the problem may not be insufficient total units, but compartmental misdistribution.

These anatomical considerations have encouraged the use of ultrasound as a selective adjunct to masseter assessment and injection planning. Ryoo and colleagues prospectively investigated an ultrasound-guided targeted injection approach for lower-face contouring, while other anatomical and imaging studies have described regional variations in muscle architecture and the potential relevance of the deep inferior tendon [16,17,18]. Ultrasound may be particularly informative when the muscle boundaries or compartments are uncertain, when paradoxical bulging has occurred, or when substantial anatomical changes are suspected after repeated treatment. However, the available evidence does not establish that ultrasound guidance is necessary for every routine masseter injection or that one injection pattern is optimal across all patients. Selection of injection sites and dose should therefore reflect the clinical objective, current anatomy, previous treatment response, and available evidence rather than a presumed universal superiority of imaging-guided treatment.

Repeated treatment safety also deserves emphasis. The 2024 randomized, triple-blind clinical trial by de Souza Nobre and colleagues showed that repeated low-dose onabotulinumtoxinA treatment reduced masseter thickness and contractility more persistently than a single session, but repeated treatment also altered masticatory performance [30]. Other papers have raised concern about mandibular bone changes after recurrent injections [15]. These findings do not negate the aesthetic utility of masseter treatment, but they reinforce the need for dose restraint over time. A high initial dose in a very thick masseter may be justified; a high maintenance dose after several successful cycles may not be.

For masseter contouring, the literature provides more direct clinical evidence relating treatment to measurable changes in muscle thickness and facial contour than is available for many smaller facial targets [15,16,17,18,27,28,29,30]. Nevertheless, the studies differ in BoNT-A formulation, dose reporting, injection distribution, patient selection, and outcome assessment. The approximately 20–40-unit-per-side onabotulinumtoxinA-oriented range presented in Table 1 is therefore an authors’ clinical synthesis rather than a single prospectively validated starting-dose interval. It should not be inferred by numerical conversion from studies using other BoNT-A formulations [1,27,33]. Treatment planning should consider baseline prominence, contraction pattern, muscle architecture, functional status, previous treatment, and the desired degree of contour change. At subsequent sessions, the original dose should be reassessed rather than automatically repeated, particularly when muscle size or masticatory function has changed [28,30]. Ultrasound may support this reassessment in selected cases but should not be presented as a universal requirement.

In short, the masseter is the facial muscle in which a dose–thickness relationship is most defensible. Yet even here, the relationship is not a crude linear formula. It is mediated by muscle architecture, compartmentalization, treatment history, ultrasound findings, and the desired degree of aesthetic change. The lesson is not simply that thicker muscles need more toxin. It is that thick muscles make anatomy visible in a way that exposes the limitations of one-size-fits-all dosing.

The temporalis is not a routine aesthetic wrinkle target, but it is relevant to facial contouring because of its role in mastication and its anatomical and functional relationship with the masseter. Published reports of BoNT-A treatment for temporalis hypertrophy are limited primarily to individual cases and small clinical descriptions [49,50,51]. These reports demonstrate the feasibility of treatment in selected circumstances but do not establish a standardized aesthetic indication, an optimal injection pattern, or a validated starting-dose range. In particular, reported doses must be interpreted according to the specific BoNT-A formulation used, the treated side or sides, and the underlying clinical indication. Accordingly, Table 1 does not propose a universal aesthetic dose for the temporalis. The limited evidence supports individualized assessment rather than routine extrapolation from masseter contouring protocols.

The temporalis also warrants attention during follow-up after masseter treatment. A randomized, triple-blind clinical study reported changes in anterior temporalis thickness, electromyographic activity, and pain-related outcomes following repeated BoNT-A treatment of masseter hypertrophy [52]. These observations suggest that repeated weakening of one masticatory muscle may be accompanied by functional or morphological changes in another, although the available evidence does not establish that such changes occur uniformly or require preventive temporalis injection. Clinical reassessment should therefore consider temporal fullness or hollowing, masticatory discomfort, asymmetry, muscle recruitment, and the cumulative treatment history. Ultrasound may be useful when objective evaluation of muscle thickness or architecture could influence management, but it should remain a selective adjunct rather than a mandatory component of every follow-up visit. In the absence of a standardized aesthetic dose and robust comparative evidence, temporalis treatment should be considered separately from routine masseter contouring, with particular attention to functional consequences and the risk of excessive muscle reduction.

### 2.15. Parotid and Submandibular Glands

Although this review is organized mainly by muscle, the parotid and submandibular glands deserve inclusion because BoNT is increasingly used for salivary reduction, glandular hypertrophy, and lower/lateral facial contouring. These targets differ mechanistically from facial muscles: the clinical effect is reduction in parasympathetic glandular secretion and, over time, reduction in glandular prominence rather than chemodenervation of a contracting expression muscle [31,32]. Therefore, gland “dose” should not be inferred from muscle thickness alone.

For the submandibular gland, an ultrasound study involving 42 participants, supplemented by cadaveric anatomical assessment, reported variation in gland depth, thickness, and relationships with nearby vascular structures [31]. These findings are relevant to anatomical localization and the avoidance of unintended injection into adjacent tissues. However, measurements of gland thickness and skin-to-gland distance do not independently establish an optimal BoNT-A dose or demonstrate that ultrasound guidance improves clinical outcomes in every patient. The anatomical recommendations described in this study should therefore be distinguished from evidence derived from prospective therapeutic dose-finding trials. Ultrasound may be particularly useful when gland boundaries are difficult to identify, anatomical variation is suspected, or the proximity of vascular and swallowing-related structures raises procedural concern. Potential complications include bruising, xerostomia, dysphagia, and unintended effects on adjacent muscles. Accordingly, Table 1 does not assign a universal aesthetic starting dose to the submandibular gland, and ultrasound is presented as a selective anatomical adjunct rather than a mandatory component of all glandular treatment.

For the parotid gland, an ultrasound-based anatomical investigation involving 40 participants described the gland’s position relative to the masseter, parotideomasseteric fascia, and surrounding structures [32]. The study reported regional measurements of skin-to-gland distance, gland thickness, and anterior extension, providing an anatomical rationale for more precise localization during procedures involving the parotid region. The proposed injection depth and surface landmarks in that publication should be interpreted as anatomy-based technical guidance rather than as a clinically validated universal injection protocol. In particular, the study does not establish a dose–response relationship between parotid thickness and BoNT-A requirements. Consideration of the parotid duct, superficial vessels, and facial nerve branches remains important when evaluating procedural risk. Ultrasound can assist with localization in selected cases, especially when surface landmarks are unreliable or previous treatment has altered regional anatomy, but its routine use cannot be mandated on the basis of anatomical evidence alone [32].

The practical conclusion for both glands is that treatment planning should be indication-specific, anatomically informed, and attentive to functional safety. Unlike skeletal muscle contouring, glandular treatment involves modulation of secretion and, in selected contexts, glandular prominence; therefore, muscle-derived dosing assumptions should not be transferred directly to these targets. The available anatomical studies provide useful localization information but do not establish standardized aesthetic BoNT-A starting doses for either gland [31,32]. The evidence supporting treatment for salivary disorders should also be distinguished from evidence supporting elective facial contouring, because the objectives, patient populations, and acceptable risk profiles differ. When glandular treatment is considered, potential adverse effects, including xerostomia, dysphagia, bruising, and unintended effects on adjacent structures, should be assessed individually. Ultrasound may assist selected procedures or reassessments when anatomical uncertainty could affect management, but should not be characterized as universally necessary. Table 1 therefore records the absence of an established aesthetic starting dose rather than proposing an unsupported numerical regimen.

## 3. Discussion

### 3.1. Do Dose and Muscle Thickness Correlate Across Facial Muscles?

The principal conclusion of this narrative review is that muscle thickness may inform facial BoNT-A treatment planning, but its relationship with dose is target-dependent and cannot be expressed as a universal dosing rule. The clearest clinical relevance of muscle size is found in contouring indications involving the masseter, where prospective, randomized, and observational studies have examined changes in muscle thickness alongside facial contour outcomes [15,16,17,18,27,28,29,30]. Even in this region, however, the evidence does not establish a universally applicable formula linking baseline thickness to the optimal number of toxin units. Formulation, injection distribution, muscle architecture, treatment history, and the desired clinical effect remain important considerations [1,16,17,18,27,33]. For smaller facial expression muscles, anatomical dimensions are only one component of treatment planning, and functional consequences may place greater constraints on dose selection than muscle bulk itself. Thus, muscle thickness should be regarded as a potential anatomical modifier rather than an independently validated dosing determinant.

At the other end of the spectrum are muscles such as the orbicularis oris, DAO, DLI, nasalis, and lateral orbicularis oculi. In these regions the absolute muscle thickness is small, the functional consequences of diffusion are significant, and clinical response depends more on vector control and local anatomy than on measurable bulk [9,10,23,24,45,46]. A thicker DAO or stronger orbicularis oris does not invite proportional dose escalation in the way a hypertrophic masseter might. Instead, the practitioner is constrained by the adverse-effect threshold. In such muscles, thickness has at most a secondary role. It may help the injector choose the upper or lower end of a narrow dose band, but it does not create a fundamentally different dosing category.

The frontalis and glabellar complex occupy an intermediate position. Ultrasound studies clearly show that these muscles vary by sex, BMI, age, and laterality [7,34]. Men often have thicker or stronger upper-face muscles and in practice often receive somewhat higher doses [5,7,34]. Nevertheless, the literature does not support a simple rule that dose should scale linearly with ultrasound thickness in the upper face. The reason is functional. The frontalis is an elevator whose weakening can lower the brow, while the corrugator–procerus complex sits in intimate relationship to the levator palpebrae, eyebrow position, and regional fascial compartments [4,5,8,20,21,39,40,41,54]. Therefore, dose is bounded by what the face can safely tolerate, not merely by what the muscle measures.

For clinical interpretation, the reviewed targets may be organized into three conceptual categories. The first comprises thin, functionally sensitive muscles, such as the orbicularis oris and selected periocular muscles, in which preservation of normal movement strongly constrains treatment intensity [9,45,46,47]. The second includes focal expression-vector muscles, such as the corrugator, procerus, DAO, and mentalis, where anatomical localization, contraction pattern, and interactions with adjacent muscles are central to treatment planning [4,21,22,23,24,25]. The third includes larger contouring or distributed treatment targets, particularly the masseter and platysma, where muscle architecture, treatment area, and the intended aesthetic outcome may influence injection distribution and total dose [15,16,17,18,26,27,28,29,30,37]. These categories are proposed by the authors as an interpretive framework, not as a validated classification system or a quantitative dose-selection algorithm. Moreover, the platysma and masseter should not be assumed to share the same dose–thickness relationship simply because both involve contouring. The available evidence supports a target-specific approach rather than a fixed hierarchy in which thickness automatically determines dose.

This framework also helps resolve why some clinicians feel that muscle thickness “matters a lot” while others believe it “does not matter much.” Both impressions are correct within their respective anatomical settings. A practitioner focused on masseter hypertrophy or platysmal banding will see a strong association between anatomy and dose. A practitioner focused mainly on the upper face will conclude that line pattern, muscle strength, and complication avoidance matter more than thickness. The real synthesis is that facial BoNT-A dosing is anatomy responsive, but different muscles express anatomy through different dominant variables.

These nonstandard targets further clarify the limits of a thickness-only model. Temporalis bulk may be clinically important, but it can reflect primary hypertrophy or compensatory recruitment after masseter treatment; therefore, thickness must be interpreted with masticatory function and symptoms [49,50,51,52]. The parotid and submandibular glands are glandular rather than contractile targets, so thickness and depth are mainly used to localize the safe injection plane and estimate diffusion risk, not to justify muscle-like dose escalation [31,32].

### 3.2. Would Neuromuscular Junction-Targeted or Highly Distributed Injection Help with Fewer Units?

**Figure 2:**
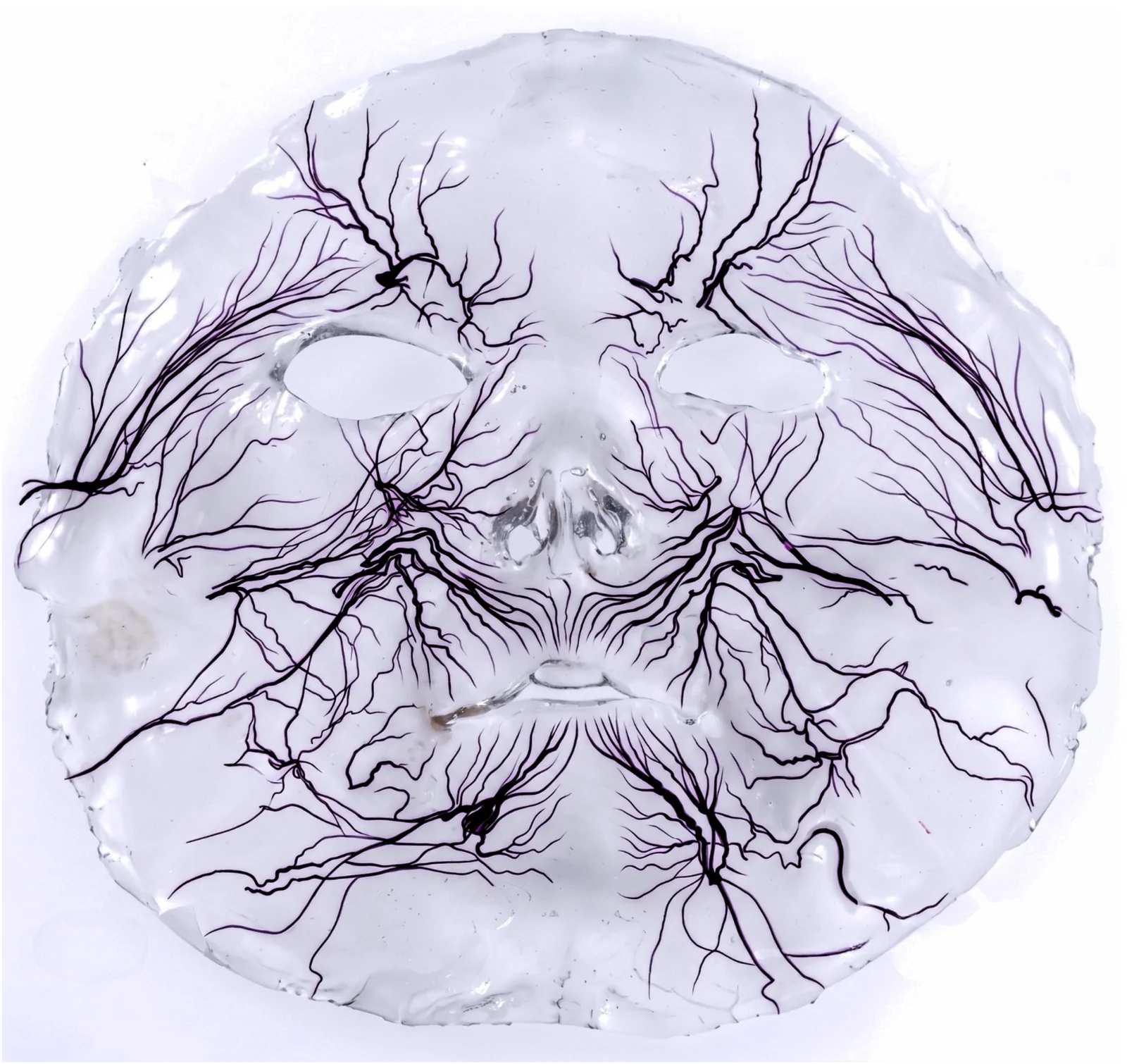
Schematic image of the neural distribution of the facial areas.

Neuromuscular junction-targeted injection has a plausible anatomical and pharmacological rationale, but whether it consistently permits lower total BoNT-A doses in facial aesthetic treatment remains uncertain. BoNT-A acts at presynaptic cholinergic nerve terminals, and injection placement relative to motor endplate regions may influence the resulting muscular effect [2,3,11,12,13]. Lapatki and colleagues demonstrated that injection location relative to the endplate zone affected treatment response in a human experimental model [13], while evidence from therapeutic neuromuscular applications also supports the relevance of anatomical targeting [12]. However, findings from experimental and non-aesthetic settings cannot be assumed to establish an equivalent dose-sparing effect across facial muscles. Differences in muscle architecture, treatment objectives, injection volume, and functional sensitivity may modify the clinical response. Thus, motor-zone targeting should be presented as an evidence-informed anatomical principle rather than as a validated method for universally reducing aesthetic BoNT-A dose (Figure 2).

Clinical and anatomical studies provide varying degrees of support for more precise injection planning. A randomized study of ultrasound-guided glabellar injection reported differences in treatment outcomes compared with conventional injection [35], while anatomical investigations of frontalis innervation and platysma motor-nerve distribution have identified features potentially relevant to injection placement [20,36,54]. Masseter imaging studies similarly describe muscle compartments and tendon-related anatomy that may help explain variable treatment responses or paradoxical bulging [16,17,18]. Nevertheless, these findings address different clinical questions and should not be pooled as proof that imaging or motor-zone targeting consistently reduces the required toxin dose. The ultrasound literature is also heterogeneous in indication, technique, and outcome assessment [19,34]. Ultrasound may be useful when anatomical uncertainty, prior treatment, or a specific complication makes additional visualization clinically relevant, but the available evidence does not justify presenting it as mandatory for every routine facial BoNT-A injection. The potential benefits of targeted injection should therefore be distinguished from direct evidence of dose reduction.

The term “highly distributed injection” requires careful definition. Dividing a total dose among multiple anatomically selected sites may help address the spatial distribution of activity in broad or structurally complex muscles, but the optimal number and location of injection points depend on the target and indication [16,17,20,26,36,37]. A distributed pattern should not be assumed to produce greater efficacy, improved safety, or lower total dose without comparative evidence. Injection volume, concentration, and local tissue characteristics can influence toxin spread, as demonstrated in experimental diffusion research [14]. Consequently, anatomical targeting and dose distribution should be evaluated as related but distinct treatment variables. The aim is to achieve the intended clinical effect while limiting unintended weakness, rather than to maximize the number of injection sites or presume that smaller aliquots are inherently superior.

The available evidence supports anatomical precision as a reasonable treatment-planning objective, but does not establish a consistent or quantifiable reduction in total BoNT-A units through motor-zone targeting or distributed injection. More accurate localization may improve the correspondence between injection placement and the intended anatomical target, while imaging may assist selected procedures involving uncertain anatomy or previous treatment-related changes [16,19,20,35,36,54]. However, evidence of improved localization or clinical response is not equivalent to evidence that a lower total dose achieves comparable efficacy and duration. Demonstrating a true dose-sparing effect would require controlled comparisons using the same BoNT-A formulation, prespecified dose levels, standardized injection patterns, and clinically meaningful outcomes. Until such evidence is available, reduced-dose approaches should be regarded as individualized clinical proposals rather than established consequences of neuromuscular junction targeting.

For glandular targets, the relevant anatomical objective differs from motor-endplate targeting in skeletal muscle. The parotid and submandibular glands require attention to gland boundaries, adjacent vessels, ducts, facial nerve branches, and structures involved in oral and swallowing function [31,32]. Ultrasound-based anatomical studies may inform localization and procedural planning, but they do not establish that imaging guidance is necessary for every glandular injection or that it permits a lower effective BoNT-A dose. Glandular treatment should therefore be evaluated according to its specific indication, functional risks, and available clinical evidence, without extrapolating muscle-based dose-sparing assumptions to secretory tissue.

### 3.3. Muscle-Specific Dose Directions for Clinical Practice

Because the available literature is heterogeneous, muscle-specific dosing recommendations should be interpreted according to their indication, formulation, and level of supporting evidence. Regulatory dosing regimens, randomized or prospective clinical findings, observational reports, and expert-derived recommendations do not carry equivalent evidentiary weight. Table 1 therefore distinguishes these categories rather than presenting all numerical ranges as equally established. In particular, BoNT-A units are not interchangeable between formulations, and findings obtained with one product should not be converted into another product’s dosing recommendations without appropriate validation [1,5,33]. The following clinical considerations are intended as an evidence-informed interpretive framework, not as a validated dose-selection algorithm.

First, for relatively thin or functionally sensitive muscles, treatment planning should prioritize preservation of normal movement and adjacent muscle function rather than rely primarily on thickness. In the frontalis, baseline eyebrow position, compensatory recruitment, forehead dimensions, and the intended degree of wrinkle reduction are important considerations [4,20,40]. For the lateral orbicularis oculi, the approved onabotulinumtoxinA regimen provides a regulatory reference, whereas alternative individualized approaches should be interpreted according to their supporting evidence [9,55]. Medial periocular and perioral applications require additional caution because relatively small changes in muscle activity may affect eyelid support, blinking, lip movement, speech, or oral competence [45,46,47]. No general numerical dose adjustment based on muscle thickness alone can be established for these targets from the available evidence.

Second, for focal expression-vector muscles such as the corrugator, procerus, depressor anguli oris, and mentalis, the direction and distribution of muscular activity are central to treatment planning. The approved onabotulinumtoxinA glabellar regimen provides a defined regional reference for the corrugator–procerus complex [8,55], whereas DAO and mentalis recommendations rely more heavily on anatomical investigations, procedural reports, and expert guidance [6,21,22,23,24,25]. These differences in evidence strength should remain explicit. Apparent muscle prominence or stronger contraction may justify closer anatomical assessment, but neither finding establishes a validated rule for increasing dose by a specified number of units. Individualized treatment should also account for baseline asymmetry and the potential consequences of unintended weakness in adjacent muscles.

Third, for larger contouring or distributed treatment targets, the masseter and platysma require separate consideration because their treatment objectives and supporting evidence differ. Masseter contouring studies have examined changes in muscle thickness and facial shape, but differences in formulation, injection pattern, and study design limit the derivation of a universal thickness-based dose rule [15,16,17,18,27,28,29,30]. Repeated masseter treatment may alter muscle dimensions and function, making reassessment of the current anatomy and previous response important before selecting subsequent doses [28,30]. For platysma bands, a phase 3 randomized study and regulatory prescribing information provide evidence for defined onabotulinumtoxinA treatment regimens [37,55]. These regimens should not be generalized to all jawline-lifting techniques or other formulations. In both regions, injection distribution and total dose should reflect the specific indication, relevant evidence, anatomical findings, and functional safety rather than an assumption that greater muscle size necessarily requires proportionally more toxin.

Less standardized targets require particular caution when translating anatomical descriptions into dosing recommendations. Depressor supercilii treatment and medial orbicularis oculi microinjection represent anatomically sensitive, individualized applications for which universally validated muscle-specific starting doses are lacking [21,47]. Temporalis treatment for contour-related indications is supported primarily by limited clinical reports, and the available literature does not establish a standardized aesthetic dosing regimen [49,50,51,52]. Similarly, anatomical ultrasound studies of the parotid and submandibular glands inform localization and procedural risk assessment but do not independently establish optimal aesthetic BoNT-A doses [31,32]. These targets should therefore be discussed separately from approved cosmetic indications, with explicit attention to functional consequences, anatomical uncertainty, and the limitations of the evidence. Ultrasound may be useful in selected patients when additional anatomical information could influence treatment planning or reassessment, but it should not be presented as a universal prerequisite for injection.

### 3.4. Integrating Muscle Thickness into a Proposed Clinical Decision Framework

Muscle thickness can be incorporated into a proposed clinical decision framework without treating it as an independent determinant of BoNT-A dose. The initial consideration is the treatment indication, including whether the objective is reduction in dynamic wrinkles, modification of facial expression, muscle contouring, or modulation of glandular secretion. The target’s functional role, contraction pattern, adjacent anatomy, previous treatment history, and potential consequences of unintended weakness should then be assessed. Clinical examination and palpation are relevant to this assessment, while ultrasound may provide additional anatomical information in selected cases involving deep, complex, or uncertain structures [7,19,34]. Injection distribution and formulation-specific dosing should subsequently be considered in relation to the available regulatory and clinical evidence. This sequence is an authors’ proposed interpretive framework, not a prospectively validated dosing algorithm.

The available evidence does not support a fixed numerical increment in BoNT-A dose for a given increase in muscle thickness. In smaller facial expression muscles, changes in apparent muscle prominence should be interpreted alongside contraction strength, functional requirements, and the risk of unintended effects on adjacent structures [4,9,21,22,23,24,25]. For masseter contouring, baseline muscle size and architecture may be relevant to treatment planning, but published studies do not establish a universally applicable relationship between each additional millimeter of thickness and a specified increase in toxin units [15,16,17,18,27,28,29,30]. Furthermore, repeated treatment may alter muscle dimensions, making reassessment more appropriate than automatic repetition or escalation of an earlier regimen [28,30]. Numerical ranges in Table 1 should therefore be interpreted according to their evidence category and BoNT-A formulation rather than used as thickness-based escalation rules [1,33].

Preservation of natural expression and regional function is an important consideration in aesthetic BoNT-A treatment, although these outcomes are not measured consistently across the studies included in this review. Facial muscles operate as interacting functional units, and excessive weakening of an individual target may alter the balance of adjacent muscles. Consequently, treatment planning should consider muscle thickness together with contraction vectors, treatment field, anatomical relationships, previous response, and patient-specific functional risks. This approach is clinically plausible and consistent with the anatomical literature, but its superiority over other dosing strategies has not been established through standardized comparative trials. It should therefore be regarded as a cautious clinical interpretation rather than a proven method for optimizing aesthetic outcomes.

Table 2 summarizes the proposed clinical considerations for different anatomical targets, distinguishing potential roles of thickness and imaging from unvalidated assumptions about dose reduction.

### 3.5. Safety, Limitations, and Future Directions

Facial BoNT-A treatment has a generally favorable safety profile in appropriately selected patients, but adverse events vary by indication, target anatomy, formulation, and injection technique. A meta-analysis involving 9669 patients provides an overview of adverse events associated with aesthetic facial treatment [38], while a systematic review of upper-face complications identifies events including eyelid ptosis, eyebrow changes, headache, and localized muscular effects [39]. Lower-face and neck applications introduce additional functional concerns, including smile asymmetry, impaired lip competence, speech changes, dysphagia, and altered mastication [6,22,23,24,25,26,30,36,37,45,46]. These complications should not be attributed solely to anatomical misplacement: dose, diffusion, patient susceptibility, previous treatment, and treatment indication may also contribute. Safety assessment should therefore consider both the intended aesthetic benefit and the potential functional consequences of weakening the target or adjacent structures.

Less standardized targets warrant particular attention because the available evidence may be predominantly anatomical or derived from small clinical reports. Medial orbicularis oculi and depressor supercilii injections involve functionally sensitive periocular structures, where unintended diffusion or excessive weakening may affect eyelid position and periocular function [21,47]. Parotid and submandibular gland treatment requires a different safety assessment, including the possibility of xerostomia, dysphagia, bruising, and effects on adjacent neurovascular or muscular structures [31,32]. Anatomical ultrasound studies can help identify gland boundaries and regional relationships, but they do not by themselves establish the comparative safety or efficacy of ultrasound-guided versus landmark-guided injection. Ultrasound should therefore be considered selectively when additional anatomical information is likely to affect procedural planning, rather than characterized as a universal requirement. Cosmetic glandular contouring must also be distinguished from treatment of salivary disorders because the intended benefits and acceptable risk–benefit balance differ.

Several limitations constrain both the underlying evidence and the conclusions of this review. Studies differ in BoNT-A formulation, dilution, concentration, injection distribution, clinical indication, outcome definition, and follow-up duration. Reported doses may refer to individual sites, one side of the face, or an entire treatment region, limiting direct comparison. Evidence strength is also uneven across targets: certain approved aesthetic indications are supported by regulatory studies and randomized trials, whereas many lower-face, temporalis, and glandular applications rely primarily on anatomical investigations, observational reports, case studies, or expert consensus. Multi-muscle indications further complicate attribution of outcomes to a single muscle or dose. Although ultrasound can quantify selected anatomical features, relatively few clinical studies prospectively test whether baseline muscle thickness predicts an optimal dose [7,15,16,17,18,27,28,29,30,34,35]. Product-specific units are not interchangeable [1]. Accordingly, the evidence categories in Table 1 should be interpreted as a qualitative hierarchy of source types, not as formal certainty ratings or proof that a proposed starting range is clinically validated.

This manuscript should be interpreted as a structured narrative review and clinical synthesis, not as a systematic review or meta-analysis. Although the literature search and study selection were organized around predefined clinical questions, the review did not undertake a comprehensive systematic-review process, formal study-level risk-of-bias assessment, or quantitative evidence synthesis. Consequently, relevant studies may have been missed, and the included evidence is susceptible to selection, reporting, and publication biases. The substantial heterogeneity in anatomical targets, formulations, indications, and outcome measures further limits direct comparison of dosing regimens. The proposed clinical framework and authors’ synthesis ranges should therefore be regarded as interpretive guidance requiring prospective validation, rather than as independently established treatment standards.

A major research priority is the development of prospective, formulation-specific dose-comparison studies with standardized efficacy and safety outcomes. For the frontalis, glabellar complex, mentalis, and depressor anguli oris, studies could evaluate whether baseline muscle thickness, contraction characteristics, or anatomical distribution predict treatment response beyond conventional clinical assessment. For the masseter, prospective investigations should examine the relationships among baseline thickness, compartmental anatomy, previous treatment exposure, retreatment intervals, facial contour outcomes, and masticatory function [16,17,18,27,28,29,30]. Repeated-treatment studies should specifically determine whether changes in muscle dimensions warrant dose adjustment, rather than assuming that the initial regimen remains appropriate. For the platysma and other broad targets, controlled comparisons of established injection patterns and anatomically informed alternatives could clarify whether targeting improves outcomes or safety [19,36,37,48]. Any proposed dose-sparing effect should be tested directly using the same formulation and clinically comparable treatment endpoints.

A second research priority is to establish when ultrasound provides clinically meaningful additional information in aesthetic BoNT-A treatment. A systematic review addressing ultrasound guidance in facial muscles innervated by the facial nerve summarizes an emerging but heterogeneous evidence base [19]. However, the reviewed literature does not establish that ultrasound is necessary for all routine injections or that imaging consistently improves clinical outcomes or reduces toxin requirements across indications. Future comparative studies should identify the anatomical or clinical circumstances in which ultrasound changes treatment decisions, such as uncertain muscle boundaries, complex masseter architecture, previous treatment-related changes, or selected glandular procedures. Standardized reporting of muscle thickness, injection depth, formulation, dose, clinical outcomes, and adverse events would help determine whether imaging-based planning provides measurable advantages over conventional assessment. Until then, ultrasound is best described as a selective adjunct rather than a universal component of facial BoNT-A treatment.

Long-term treatment effects also require closer investigation. Observational studies of repeated BoNT-A treatment suggest that clinical response and retreatment patterns may evolve over time, although findings vary by anatomical region and indication [28,30,53]. In masseter contouring, repeated treatment has been associated with changes in muscle thickness and facial contour, making reassessment of current muscle dimensions, treatment response, and masticatory function relevant before subsequent injections [28,30]. Similar caution is appropriate when considering repeated temporalis treatment, although the aesthetic evidence for this target remains limited [49,50,51,52]. These observations do not establish a universal schedule for maintenance treatment or a predictable reduction in dose over successive sessions. Prospective longitudinal studies are needed to determine how structural changes, functional outcomes, treatment intervals, and cumulative exposure should inform individualized retreatment decisions.

## 4. Conclusions

This structured narrative review does not identify a universal relationship between facial muscle thickness and the optimal dose of botulinum toxin type A (BoNT-A). Muscle size and architecture may be relevant to treatment planning, particularly in masseter contouring, but the available studies do not establish a validated thickness-based dosing equation. For facial expression muscles, treatment objectives, contraction patterns, adjacent anatomy, and preservation of normal function are often more relevant than thickness alone. The evidence supporting numerical dose recommendations varies substantially by target, from approved product-specific regimens and randomized trials to observational studies, anatomical investigations, and expert-derived guidance. These distinctions should remain explicit when translating published findings into clinical recommendations.

Anatomically informed injection placement, including consideration of motor endplate regions and muscle compartmentalization, has a plausible mechanistic basis and may assist individualized treatment planning. However, current evidence does not establish that neuromuscular junction-targeted or highly distributed injection consistently reduces the total dose required for equivalent aesthetic efficacy. Ultrasound may provide useful additional information in selected patients, particularly when anatomical boundaries are uncertain, previous treatment has altered muscle dimensions, or deeper structures are being considered. It should not be regarded as mandatory for all routine facial BoNT-A injections. Further comparative studies are needed to determine whether these approaches improve efficacy, safety, consistency, or dose efficiency.

In clinical practice, dose selection should remain formulation-specific, indication-specific, and attentive to functional safety. Approved onabotulinumtoxinA regimens provide reference points for their respective labeled indications, but these doses should not be generalized to other formulations or off-label targets because BoNT-A units are not interchangeable. The proposed framework in this review integrates clinical examination, muscle anatomy, treatment history, available evidence, and selective imaging when appropriate. It is the authors’ clinical synthesis rather than a prospectively validated dosing algorithm. Numerical starting ranges derived from expert practice or limited clinical reports should therefore be distinguished from doses established through regulatory or controlled clinical studies.

Less standardized targets, including the depressor supercilii, medial orbicularis oculi, temporalis, and parotid and submandibular glands, require particular caution because evidence for aesthetic dosing is limited or predominantly anatomical. Glandular treatment should be distinguished from skeletal muscle injection and from medically indicated treatment of salivary disorders. Repeated masseter or temporalis treatment also warrants reassessment of current anatomy, previous response, and relevant functional effects rather than automatic repetition of earlier dosing. Overall, individualized facial BoNT-A treatment is best understood as a balance among target anatomy, indication, formulation-specific evidence, patient-specific function, and procedural safety, with the limitations of the available literature clearly acknowledged.

## 5. Materials and Methods

### 5.1. Review Design and Clinical Questions

This manuscript was designed as a structured review of facial BoNT-A dosing literature with additional anatomy- and mechanism-based synthesis. The review questions were as follows: among facial muscles and selected glandular structures used in cosmetic or contouring botulinum toxin treatment, what dose ranges have been reported, which anatomical factors modify those dose ranges, how much evidence supports a relationship between muscle thickness and dose, and does targeted delivery to neuromuscular junction-rich or neural-arborization zones permit similar results with fewer units? The review focused on muscles relevant to aesthetic and contouring indications rather than on the full therapeutic literature for facial palsy or movement disorders, although selected therapeutic studies were included when they clarified injection efficiency, endplate targeting, or muscle-specific or gland-specific safety [11,12,13,19,25,31,32].

### 5.2. Information Sources and Search Strategy

A structured literature search was conducted using PubMed-indexed literature and accessible full-text publisher sources, supplemented by reference-list screening of relevant reviews and consensus publications. The search covered English-language literature available through 13 April 2026. Search terms combined “botulinum toxin” or “botulinum neurotoxin type A” with “facial aesthetics,” “dose,” “dosing,” individual target names (frontalis, corrugator, procerus, depressor supercilii, orbicularis oculi, nasalis, levator labii superioris alaeque nasi, orbicularis oris, depressor anguli oris, depressor labii inferioris, mentalis, platysma, masseter, temporalis, parotid gland, and submandibular gland), and anatomical or technical concepts including “muscle thickness,” “gland thickness,” “ultrasound,” “motor endplate,” “neuromuscular junction,” “intramuscular nerve distribution,” and “ultrasound-guided injection.” Indication-specific terms included “glabellar lines,” “crow’s feet,” “bunny lines,” “gummy smile,” “bruxism,” “masseter hypertrophy,” “platysmal bands,” “salivary gland hypertrophy,” and “sialorrhea.” Eligible sources included regulatory prescribing information, randomized and prospective clinical studies, observational studies, case reports, systematic and narrative reviews, expert consensus statements, and relevant anatomical or ultrasound investigations. Because the purpose was to integrate clinical dosing with target-specific anatomy across heterogeneous evidence types, the search and synthesis were structured but were not designed as a formal systematic review or meta-analysis.

Because many aesthetic dosing papers are dispersed across dermatology, plastic surgery, anatomy, oral–maxillofacial, and cosmetic medicine journals, the review deliberately combined indication-based searching with muscle-specific searching. For example, studies on the LLSAN, levator labii superioris, and zygomaticus minor were frequently retrieved through “gummy smile” rather than by muscle name alone, while literature relevant to masseter dosing was often indexed under hypertrophy, lower-face contouring, or bruxism [15,16,17,18,27,28,29,30,42,43]. Likewise, platysma articles appeared under platysma prominence, platysmal bands, jawline lifting, and lower-face rejuvenation [25,26,36,37].

### 5.3. Eligibility Criteria

Studies were eligible when they met at least one of the following conditions: (1) they reported clinical dosing or injection technique for a specific facial muscle or facial aesthetic indication; (2) they provided formulation-specific or muscle-specific dose recommendations in a review, guideline, consensus statement, or prescribing document; (3) they evaluated ultrasound-guided or anatomically targeted injection with implications for dose, efficiency, or safety; or (4) they investigated muscle thickness, architecture, or neural distribution in a way that could plausibly inform clinical dosing. Adult human data were prioritized. High-value cadaveric or anatomical studies were included when they directly clarified injection depth, neural arborization, compartmentalization, or surface landmarks.

Ultrasound or anatomical studies of the parotid and submandibular glands were also eligible when they provided clinically applicable depth, thickness, landmark, or safety data relevant to BoNT injection [31,32].

Studies were excluded if they dealt solely with non-facial therapeutic indications without transferable relevance to facial injection, if they were purely basic science without practical dosing implications, or if they were duplicate summaries without new data. Non-English sources without interpretable dose data were not included. Where multiple publications covered the same concept, priority was given to systematic reviews, high-quality randomized trials, recent consensus statements, or the most anatomically explicit review.

### 5.4. Data Items and Synthesis Approach

For each source, the following data items were extracted when available: toxin formulation; facial muscle or indication; total dose and/or dose per side; number of injection points; depth or tissue plane; indication for treatment; whether the technique was landmark-based, anatomy-guided, or ultrasound-guided; and any discussion of modifying factors such as sex, muscle strength, facial morphology, severity of contraction, muscle thickness, or prior response. Attention was paid to whether studies described units in botulinum toxin A terms or in another product’s native units, because unit non-interchangeability is central to any meaningful dose comparison [1,5,33]. For glandular targets, extracted data additionally included skin-to-gland depth, gland thickness, surface landmark, proximity to vessels or ducts, and whether ultrasound guidance was recommended.

The planned synthesis was qualitative rather than meta-analytic. A quantitative pooled analysis was not appropriate because the literature contains several sources of irreducible heterogeneity: different toxins, different dilution schemes, different outcome measures, different treated populations, different follow-up intervals, and inconsistent reporting of whether doses are per point, per muscle, per side, or total face. The same aesthetic indication may also be treated through different muscles depending on facial subtype. For example, gummy smile may be treated through a Yonsei-point strategy, an LLSAN-dominant strategy, or a multi-point elevator-complex approach [42,43,44].

To prevent the conflation of regulatory recommendations, experimentally evaluated doses, and expert-derived clinical suggestions, dose information was organized into four evidence categories: (1) doses specified in regulatory prescribing information for a particular formulation and approved indication; (2) doses evaluated in randomized or prospective clinical investigations; (3) doses reported in observational studies or case reports; and (4) authors’ evidence-informed synthesis or proposed starting ranges. These categories are presented separately in Table 1. A regulatory dose assigned to a treatment region was not interpreted as a separately approved dose for each constituent muscle. Similarly, doses reported per injection point, per side, or as a bilateral total were distinguished wherever the source permitted. Product-specific potency units were retained without assuming numerical interchangeability among BoNT-A formulations [1,5,33]. Where a specific evidence category was not established by the sources considered, no numerical dose was inferred from another category. Authors’ proposed ranges were interpreted as contextual clinical syntheses rather than independently validated dose recommendations.

### 5.5. Assessment of Evidence Quality and Bias

The evidence base included heterogeneous study designs, ranging from randomized clinical trials and prospective investigations to observational reports, expert consensus statements, anatomical studies, and ultrasound-based investigations. Accordingly, evidence was assessed through a structured qualitative appraisal rather than a pooled quantitative estimate or a uniform numerical risk-of-bias score. Clinical studies were considered in relation to study design, presence of a comparator, sample characteristics, formulation-specific dose reporting, outcome assessment, follow-up duration, and relevance to the anatomical target and indication under review. Particular attention was given to potential selection bias in uncontrolled studies, performance and detection bias in nonblinded assessments, confounding by baseline muscle activity or previous treatment, and incomplete reporting of dose distribution or treatment outcomes.

For interpretation, randomized and well-controlled prospective clinical investigations were given greater weight for claims about efficacy and dose–response, while observational studies and case reports were treated primarily as supportive or hypothesis-generating evidence. Regulatory prescribing information was distinguished from clinical-study evidence because approval applies to specified formulations, indications, and administration regimens. Anatomical and ultrasound studies were used to support target localization, tissue characteristics, and plausible safety considerations, but were not treated as direct proof of superior clinical outcomes or lower dose requirements. Expert consensus recommendations were considered useful for clinical context but were not equated with independently validated dose–response relationships.

The principal limitations affecting certainty of interpretation were heterogeneity in toxin formulations and dosing units, inconsistent reporting of per-point versus per-side or total doses, variation in injection technique and outcome definitions, small or uncontrolled study populations for several off-label targets, and limited direct comparisons of anatomy-guided versus conventional treatment. Furthermore, evidence derived from functional or therapeutic indications was not assumed to establish efficacy or safety for aesthetic contouring. These limitations informed the evidence-stratified presentation in Table 1 and the cautious interpretation of authors’ proposed starting ranges. Because a complete study-level assessment using a validated risk-of-bias instrument was not performed, the appraisal should not be interpreted as a formal GRADE certainty assessment or a validated risk-of-bias evaluation.

### 5.6. Reporting Philosophy

Because the review is clinically oriented and because the literature often mixes muscle names, aesthetic regions, and injection patterns, the results are reported in a way that serves clinical decision-making. The Section 2 is organized by muscle, muscle complex, accessory masticatory target, or glandular target. Each subsection addresses: what the muscle does aesthetically; what dose ranges are reported; what anatomical or functional factors change those ranges; how much thickness likely matters; and whether distributed, NMJ-targeted, or ultrasound-guided glandular injection could plausibly reduce the number of units needed or reduce off-target diffusion. A separate integrative discussion then examines cross-cutting concepts such as dose–response, product non-interchangeability, muscle thickness, ultrasound, and safety.

Because glandular targets and accessory masticatory muscles do not follow the same dose logic as thin mimetic muscles, they are discussed as separate anatomical categories rather than being forced into a single wrinkle-based dosing model.

## Figures and Tables

**Table 2:** Proposed anatomical and functional considerations for individualized BoNT-A treatment planning. This framework is an authors’ synthesis and has not been prospectively validated as a dosing algorithm.

Target Category	Examples	Relevance of Thickness	Treatment-Planning Considerations and Evidence Limitations
Thin, functionally sensitive muscles	Frontalis; orbicularis oris	One of several anatomical considerations; not an established independent dose predictor	Prioritize eyebrow position, expression, oral competence, and adjacent-muscle effects. No validated thickness-based dose adjustment.
Focal expression-vector muscles	Corrugator/procerus; DAO; mentalis	May contribute to clinical assessment; quantitative relationship with dose is uncertain	Consider contraction direction, regional anatomy, and formulation-specific evidence. Motor-zone targeting has not demonstrated a universal dose-sparing effect.
Broad superficial muscle	Platysma	Treatment field and active-band pattern may be more relevant than thickness alone	Distinguish labeled platysma-band treatment from other off-label neck and jawline techniques. Avoid extrapolating approved doses to different indications.
Bulky contouring muscle	Masseter	Clinically relevant to contour assessment; no universal dose–thickness equation	Evaluate prominence, architecture, prior treatment, and masticatory function. Ultrasound may help in selected cases. Reassess before repeated treatment.
Medial periocular and glabellar refinement targets	Depressor supercilii; medial orbicularis oculi	Limited evidence as a quantitative dosing determinant	Functional sensitivity and proximity to adjacent structures favor cautious, individualized planning. Standardized target-specific doses are lacking.
Accessory masticatory muscle	Temporalis	May inform assessment of hypertrophy or treatment-related changes	Evidence for aesthetic dosing is primarily case-based. Consider masticatory function, asymmetry, and previous masseter treatment. No validated starting-dose range.
Glandular targets	Parotid; submandibular gland	Relevant to localization, not an established aesthetic dose predictor	Distinguish secretory and aesthetic indications. Assess ducts, vessels, nerves, and swallowing-related structures. Ultrasound may assist selected cases; no universal aesthetic dose is established.

## Data Availability

No new data were created or analyzed in this study. Data sharing is not applicable to this article.

## Abbreviations

The following abbreviations are used in this manuscript:

BoNT-A	botulinum neurotoxin type A
DAO	depressor anguli oris muscle
DLI	depressor labii inferioris muscle
LLS	levator labii superioris muscle
LLSAN	levator labii superioris alaeque nasi muscle
NMJ	neuromuscular junction
OOc	orbicularis oculi muscle
Zmi	zygomaticus minor muscle
